# Applying valency-based immuno-selection to generate broadly cross-reactive antibodies against influenza hemagglutinins

**DOI:** 10.1038/s41467-024-44889-w

**Published:** 2024-02-12

**Authors:** Daniëla Maria Hinke, Ane Marie Anderson, Kirankumar Katta, Marlene Fyrstenberg Laursen, Demo Yemane Tesfaye, Ina Charlotta Werninghaus, Davide Angeletti, Gunnveig Grødeland, Bjarne Bogen, Ranveig Braathen

**Affiliations:** 1 K.G. Jebsen Centre for Influenza Vaccine Research, University of Oslo, Oslo, Norway; 2 Institute of Immunology (IMM), University of Oslo and Oslo University Hospital, Oslo, Norway; 3 Department of Microbiology and Immunology, Institute of Biomedicine, University of Gothenburg, Gothenburg, Sweden

**Keywords:** DNA vaccines, Influenza virus, DNA vaccines

## Abstract

Conserved epitopes shared between virus subtypes are often subdominant, making it difficult to induce broadly reactive antibodies by immunization. Here, we generate a plasmid DNA mix vaccine that encodes protein heterodimers with sixteen different influenza A virus hemagglutinins (HA) representing all HA subtypes except H1 (group 1) and H7 (group 2). Each single heterodimer expresses two different HA subtypes and is targeted to MHC class II on antigen presenting cells (APC). Female mice immunized with the plasmid mix produce antibodies not only against the 16 HA subtypes, but also against non-included H1 and H7. We demonstrate that individual antibody molecules cross-react between different HAs. Furthermore, the mix vaccine induces T cell responses to conserved HA epitopes. Immunized mice are partially protected against H1 viruses. The results show that application of valency-based immuno-selection to diversified antigens can be used to direct antibody responses towards conserved (subdominant) epitopes on viral antigens.

## Abstract (web summary)

Here the authors develop a DNA vaccine, mixing 18 hemagglutinin (HA) subtypes monovalently into heterodimeric molecules, and show that it induces broadly reactive antibodies against subdominant HA epitopes and heterologous protection against influenza A viruses in mice.

## Introduction

Seasonal influenza epidemics caused by influenza A viruses (IAVs) and influenza B viruses (IBVs) are responsible for approximately 500,000 deaths worldwide every year^1,2^. Moreover, IAVs have caused four pandemics during the last century alone^3^. The main target for neutralizing antibodies against IAVs is viral surface glycoprotein hemagglutinin (HA)^4^. There are 18 different HA subtypes^5–8^. Based on similarities in amino acid sequences, these 18 subtypes are divided into group 1 (H1, H2, H5, H6, H8, H9, H11, H12, H13, H16, H17 and H18), and group 2 (H3, H4, H7, H10, H14 and H15). At present, H1 and H3 viruses circulate in the human population and regularly cause seasonal epidemics. In addition, a number of zoonotic IAV (H5, H6, H7, H9 and H10) have caused isolated but severe disease outbreaks in humans^9^.

The adaptive immune system often responds to antigen in a hierarchical manner; highly immunogenic epitopes are immunodominant, while less immunogenic epitopes are subdominant^10^. Current influenza vaccines predominantly elicit antibodies towards immunodominant epitopes in the membrane-distal head domain of HA. Unfortunately, frequent mutations in the HA head allow viral escape from pre-existing immune responses^11–13^, necessitating annual reformulations of seasonal influenza vaccines^14^. It is therefore important to develop vaccine formats that focus B cell responses towards conserved, subdominant HA epitopes, thus enhancing the generation of broadly cross-reactive antibodies.

Human monoclonal antibodies with broad reactivity to many HAs have been isolated^15^, suggesting that it should be possible to induce broadly reactive antibodies to subdominant HA epitopes by means of innovative vaccination strategies. The main specificities of broadly neutralizing antibodies are the receptor binding site in the HA head domain^16–18^, and epitopes in the fusion peptide in the conserved stem domain^19–21^.

We have recently developed a new vaccine format based on an ACID/BASE-modified Fos-Jun zipper where bivalency versus monovalency of vaccine antigens can be controlled^22^. Using this strategy, we have shown that antigen bivalency enhances the ability of APC-targeted vaccines to induce potent antibody responses^23^. As an explanation, we hypothesized that antigen bivalency increased B cell stimulation by cross-linking B cell receptors (BCRs) in APC-B cell synapses. According to this hypothesis, if two variants of an antigen are expressed together in a heterodimer, B cells with specificity for conserved epitopes shared by the two variants should be preferentially stimulated^23^. To test this idea, we here developed a vaccine strategy where single vaccine protein heterodimers should always express two different HA subtypes (e.g. HA_x,_ and HA_y_), each in a monovalent manner. In such molecules, only conserved epitopes shared between HA_x_ and HA_y_ should be bivalent while strain-specific HA epitopes should be monovalent. This vaccination strategy should preferentially stimulate B cells (antibodies) with specificity for conserved epitopes, while monovalent strain-specific epitopes should be less immunogenic. To test if such a valency-based immuno-selection strategy could enhance induction of broadly reactive antibody responses against ordinarily subdominant HA epitopes, we have here immunized mice with mixtures of plasmids that together encoded up to 18 different HA subtypes, each HA being monovalently expressed in a large number of heterodimeric vaccine molecules.

## Results

### APC-targeted heterodimeric proteins with different HAs delivered as DNA plasmids

**Fig. 1:**
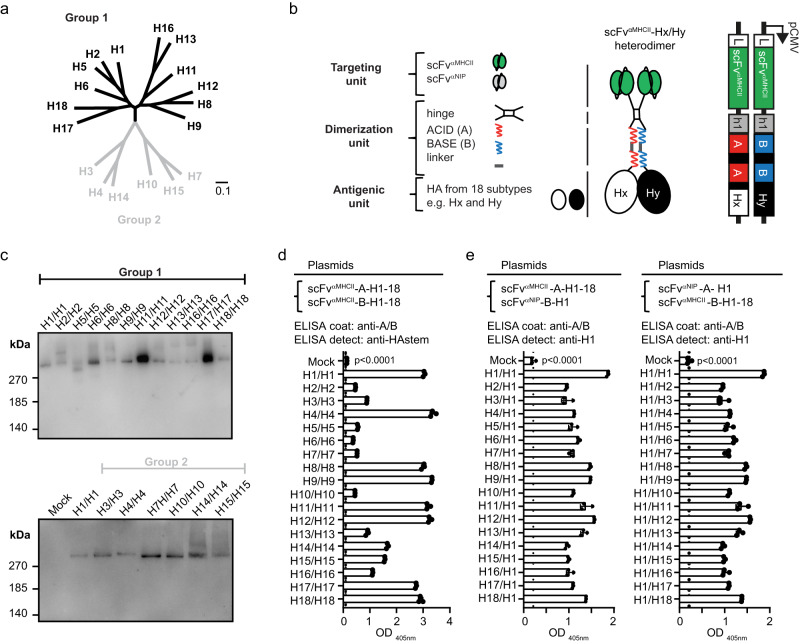
Expression of 18 different HA subtypes in heterodimeric vaccine proteins. **a** Phylogenetic tree of amino acid sequences of selected HA subtypes of group 1 (black) and group 2 (gray). Scale bar represents 10% variation in amino acid sequences. **b** Schematic representation of heterodimeric vaccine proteins (middle) and the units each chain is composed of (left). The dimerization unit contains either a duplicated ACID (A) or BASE (B) sequence of a modified Fos-Jun zipper, attached to a shortened Ig hinge that allows formation of covalent bonds. The targeting unit is either scFv of anti-MHCII (I-E^d^) mAb (scFv^αMHCII^), or scFv of anti-NIP mAb (scFv^αNIP^). The antigenic unit is any HA from subtypes H1-18 of IAV. Right: The A and the B cassette plasmids encoding the A and B chain of a heterodimer. **c**, **d** HEK293E cells were transiently co-transfected with indicated pairs of A and B plasmids that together encode heterodimers with two identical MHCII-specific targeting units and two identical HA antigenic units (HA_x_/HA_x_), for each of H1-18. Supernatants were analyzed by: (**c**) Western blot under non-reducing conditions, detected with anti-A/B mAb (*n* = 1 experiment); (**d**) sandwich ELISA employing anti-A/B and anti-HA stem mAb. **e** HEK293E cells were co-transfected with A and B plasmids that encode heterodimers with HA expression on the A and B arms as indicated (HA on A chain is indicated before the slash, followed by HA on the B chain). Left: pairing of plasmids with an HA corresponding to each of H1-18 (scFv^αMHCII^-A-H1-18) and an invariant B plasmid (scFv^αNIP^-B-H1) resulting in HA_x_/H1 heterodimers. Right: vice versa, resulting in H1/HA_x_ heterodimers. Supernatants were analyzed by sandwich ELISA using mAbs specific for A/B and H1. Shown are individual OD_405nm_ values plus mean of technical triplicates ± SD. (*n* = 1 experiment). Statistics were calculated for vaccine protein expression compared to mock, using one-way Anova with Dunnetts multiple comparisons. All heterodimeric proteins in (**d**) and (**e**) were expressed in significantly higher amounts then the mock with *p* < 0.0001. Amino acid sequences for included HA proteins in (**a**) and uncropped images for Western blots in (**c**) are provided as a Source Data file.

We selected HA variants from 18 IAV subtypes based on their commercial availability as proteins and their similarity to strains that presently circulate or have circulated in the past (Table 1). A phylogenetic tree (Fig. 1a) of selected HA subtypes was generated based on their amino acid sequences (Supplementary Fig. 1). We inserted genes for the 18 HA subtypes into A and B plasmids that together encode heterodimeric vaccine proteins due to a centrally located modified Fos-Jun zipper heterodimerization motif called A/B (ACID/BASE) (Fig. 1b)^22,24^. The different HA subtypes were expressed C-terminally of the dimerization unit. To bivalently target proteins to APC, A and B chains both expressed an N-terminal MHCII (I-E^d^)-specific scFv. In non-targeted controls a scFv specific for the hapten 4-hydroxy-3-iodo-5-nitrophenylacetic acid (NIP) replaced the MHCII-specific scFv.

**Table 1:** Overview of IAV HA subtype virus strains used in the plasmid mix vaccines, ELISA coats and challenge of mice

HA subtype	HA group	Virus strain	HA distribution on plasmids	ELISA coat	Challenge virus
H1	1	H1N1 (A/Puerto Rico/8/1934) (PR8)	B	**✓**	**✓**^**a**^
		H1N1 (A/California/07/2009) (Cal07)	B	**✓**	**✓**^**a**^
H2	1	H2N2 (A/Canada/720/2005)	B	**✓**	
H3	2	H3N2 (A/HongKong/1/1968)	B	**✓**	
H4	2	H4N6 (A/Swine/Ontario/01911-1/1999)	B	**✓**	
H5	1	H5N1 (A/Hong Kong/483/1997)	B	**✓**	
H6	1	H6N1 (A/northern shoveler/California/HKWF115/2007)	B	**✓**	
H7	2	H7N1 (A/chicken/Italy/13474/1999)	A		
		H7N1 (A/turkey/Italy/3889/1999)			**✓**^**a**^
		H7N9 (A/Shanghai/1/2013)		**✓**	
H8	1	H8N4 (A/pintail duck/Alberta/114/1979)	A	**✓**	
H9	1	H9N2 (A/Hong Kong/1073/1999)	A	**✓**	
H10	2	H10N3 (A/duck/Hunan/S11205/2012)	A	**✓**^**b**^	
H11	1	H11N2 (A/duck/Yangzhou/906/2002)	A	**✓**	
H12	1	H12N5 (A/green-winged teal/ALB/199/1991)	A	**✓**	
H13	1	H13N8 (A/black-headed gull/Netherlands/1/2000)	A	**✓**	
H14	2	H14N5 (A/Mallard/Astrakhan(Gurjev)/263/1982)	B	**✓**	
H15	2	H15N8 (A/duck/AUS/341/1983)	A	**✓**	
H16	1	H16N3 (A/black-headed gull/Sweden/5/1999)	A	**✓**	
H17	1	H17N10 (little yellow-shouldered bat/Guatemala/164/2009)	B	**✓**	
H18	1	H18N11 (A/flat-faced bat/Peru/033/2011)	B	**✓**	

^a^H1N1 and H7N1 viruses used for infection were mouse adapted.

^b^In ELISA only the HA1 subunit of H10 was used.

We first tested if all 18 HA subtypes were properly expressed in heterodimeric proteins. HEK293E cells were co-transfected with plasmid pairs that together encoded MHCII-targeted heterodimers with identical HA on their two antigenic arms. Heterodimers could be detected in supernatant by Western blot with a mAb specific for the assembled A/B heterodimerization motif (Fig. 1c), and by ELISA using a combination of mAbs against A/B and a conserved HA stem epitope (Fig. 1d). We verified the secretion of each of the 18 HA subtypes on either A or B chains by co-transfection of A plasmids expressing any of H1-18 together with B plasmids expressing H1, and vice versa. We detected similar levels of heterodimeric proteins with all MHCII-targeted plasmid combinations (Fig. 1e). The results were confirmed for non-targeted scFv^αNIP^ plasmid combinations (Supplementary Fig. 2). Expression of scFv^αMHCII^ and scFv^αNIP^, on either A or B chains, did not influence the results (Fig. 1e, Supplementary Fig. 2). These findings suggest that A and B chains were assembled and secreted as correctly folded heterodimeric vaccine proteins with any of the 18 HA subtypes as antigen.

### MHCII-targeted heterodimeric DNA vaccines that bivalently express two identical HA (H1-18) induce subtype-specific antibody responses

**Fig. 2:**
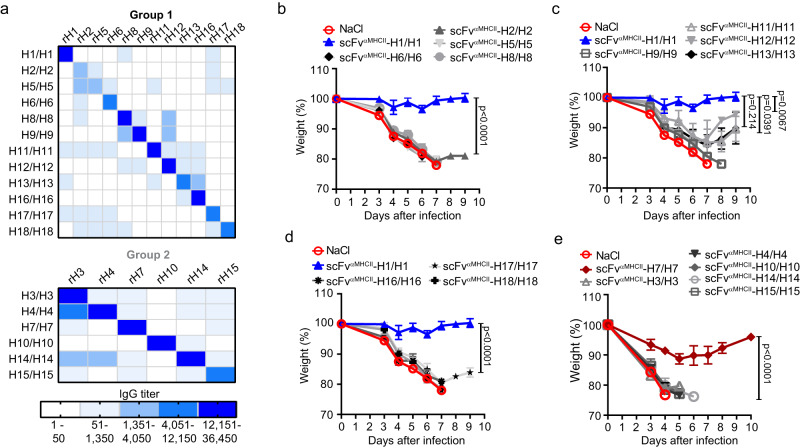
MHCII-targeted heterodimeric DNA vaccines that bivalently express two identical HA induce subtype-specific antibody responses. **a** Female BALB/cAnNRj mice (*n* = 4/group) were vaccinated i.d. with a total of 50 µg DNA (A and B plasmids) immediately followed by electroporation (EP). Plasmids encoded bivalent heterodimers with two scFv^αMHCII^ and two identical HAs for H1-18. HA subtype-specific IgG responses elicited by a given HA_x_/HA_x_ (indicated left) were analyzed six weeks after vaccination against the indicated recombinant HA (top) in ELISAs. Top matrix: group 1 HAs. Bottom matrix: group 2 HAs. Scale for heat map is indicated. **b**–**d** BALB/c mice vaccinated with indicated bivalent group 1 HAs (25 µg/plasmid, *n* = 4/group), or with scFV^αMHCII^-targeted H1/H1 (*n* = 7) or NaCl (*n* = 7) as controls, were challenged with 5xLD_50_ PR8 (H1N1) virus ten weeks after vaccination. **e** Mice vaccinated with indicated bivalent group 2 HAs (25 µg/plasmid, *n* = 4/group), or with scFV^αMHCII^-targeted H7/H7 (*n* = 4) or NaCl (*n* = 4) as controls, were challenged with 5xLD_50_ A/turkey/Italy/3889/1999 virus (H7N1). Shown are mean weight ± SEM). Mice were euthanized when they reached ≤ 80% of their original body weight and removed from the weight curves. % survival is shown in Supplementary Fig. 4. (*n* = 1 experiment). The same NaCl and H1/H1 vaccinated control mice were used in (**b**–**d**). For survival after PR8 challenge, significance was calculated on day 6 (**b**–**d**) or day 4 (**e**) for indicated groups compared to H1/H1 or H7/H7 control vaccines using one-way ANOVA with Dunnet’s multiple comparisons test. Source data are provided as a Source Data file.

BALB/c mice were vaccinated once with A/B plasmid pairs that encode MHCII-targeted heterodimers with identical HA on their two arms (i.e. bivalent expression). Plasmids were injected intradermally (i.d.) followed by electroporation (EP) to increase cellular uptake of DNA. Six weeks after vaccination, serum IgG reactivity was clearly highest against the HA subtype expressed in the vaccine (Fig. 2a). Some minor cross-reactivity was observed towards phylogenetically related HA subtypes. While most of the 18 HA subtypes induced high antibody titers, H2 and H5 were less immunogenic. The observed IgG titers correlated to the expression levels of bivalent HA vaccines (Supplementary Fig. 3). These results suggest that all 18 bivalently expressed HA subtypes are immunogenic in heterodimeric vaccine molecules.

Ten weeks after a single immunization with the various group 1 HA, mice were challenged with influenza H1N1 PR8 virus (Fig. 2b–d and Supplementary Fig. 4a–c). Vaccines expressing homologous MHCII-targeted H1/H1 from PR8 completely protected mice against PR8 virus, consistent with previous results^25^. Vaccines expressing other HA subtypes within group 1 generally failed to protect against PR8. The minor protection induced by H11, H12, and H13 could be due to T cell cross-reactivity since the antibody responses elicited by these HA subtypes poorly cross-reacted to H1 (Fig. 2a). Mice vaccinated with group 2 HA subtypes were challenged ten weeks later with H7N1 virus (A/turkey/Italy/3889/1999) (Fig. 2e and Supplementary Fig. 4d). The MHCII-targeted vaccine expressing H7/H7 (A/chicken/Italy/13474/1999 H7N1) protected against this heterologous virus strain, while vaccines with other group 2 HA subtypes did not. Overall, to obtain full vaccine-induced protection with these antigenically bivalent vaccines, the HA subtype had to match the subtype of challenging virus.

### Different HA subtypes can be combined in one heterodimeric mix vaccine

**Fig. 3:**
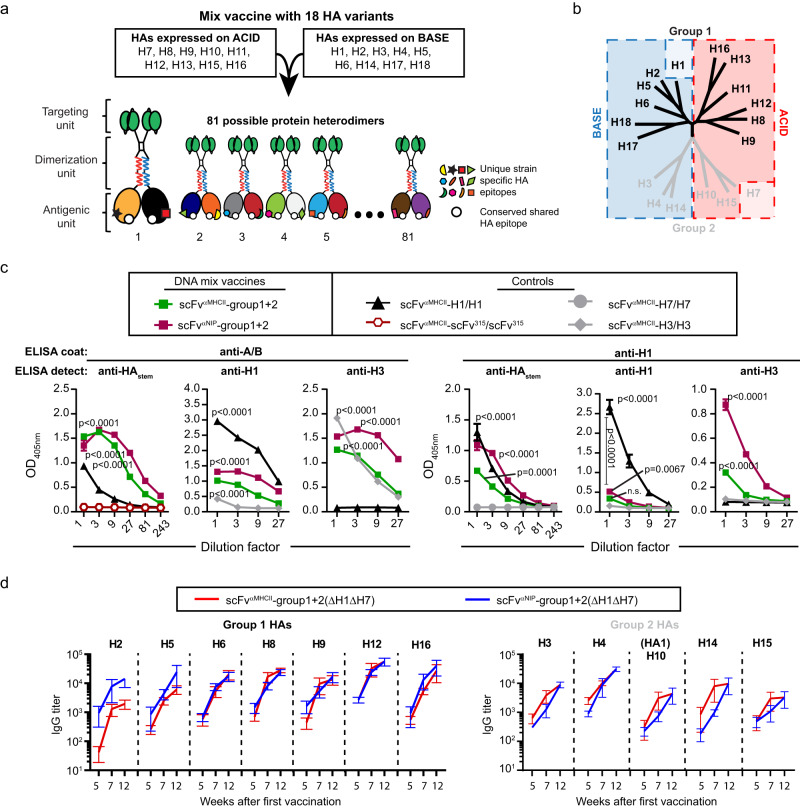
The mix plasmid vaccines are translated into vaccine proteins in vitro and induce HA subtype-specific antibody responses upon immunization of mice. **a** Co-transfection of nine A plasmids and nine B plasmids expressing the indicated 18 HA subtypes should theoretically yield 81 heterodimeric proteins. Each heterodimer should be monovalent for any HA subtype. **b** The 18 HA subtypes of group 1 and group 2 were distributed on either A or B plasmids, as indicated. In 16 HA subtype mixes, H1 and H7 (colored lightly) were excluded. **c** HEK293E cells were transiently co-transfected with the 18 HA subtype plasmid mixes (group 1 + 2, either with scFv^αMHCII^ or scFv^αNIP^) and supernatants were analyzed in sandwich ELISA with the indicated coat and detection mAbs. A/B plasmid pairs expressing bivalent HA subtypes (H1/H1, H7/H7, H3/H3) or bivalent scFv from M315 myeloma protein (scFv^315^/scFv^315^) were included as controls. Shown are mean ± SD of technical triplicates. **d** Female BALB/cAnNRj mice were immunized three times i.d./EP (week 0, 5, 10) with the 16 HA subtype mix (1 µg/plasmid), in either APC-targeted (scFv^αMHCII^) or non-targeted (scFv^αNIP^) format. Serum collected at weeks 5, 7, and 12 were analyzed by ELISA for IgG antibodies against the indicated HA subtypes encoded within the HA mix vaccine. Shown are mean IgG titers ± SEM, *n* = 6 mice/group. (*n* = 1 experiment). Statistics in (**c**) were calculated for vaccine protein expression compared to scFv^315^/scFv^315^, H3/H3, H1/H1 or H7/H7 negative controls on 1x dilution of supernatant, n.s. = not significant, one-way Anova with Dunnett’s multiple comparisons test. There were no statistical differences between targeted and non-targeted groups in (**d**) (two-tailed Mann–Whitney for each timepoint). Source data are provided as a Source Data file.

We have previously shown that antigen bivalency in dimeric vaccines increases humoral responses^23^. Heterodimers that carry two different HA subtypes are bivalent for conserved epitopes while monovalent for unique epitopes, as illustrated in Fig. 3a. We therefore hypothesized that forced monovalency for HA subtypes in dimeric vaccine molecules could selectively favor activation of B cells reactive towards shared conserved regions over those specific for unique epitopes on HA subtypes.

To test our hypothesis, we expressed nine HA subtypes with A plasmids, and the residual nine subtypes with B plasmids, equally splitting both group 1 and 2 HAs (Fig. 3b). Theoretically, 81 HA monovalent heterodimers should be formed. To test if plasmid mixtures were translated into anticipated vaccine proteins, HEK293E cells were transiently co-transfected with a mixture of the nine A plus the nine B plasmids. For both MHCII-targeted and NIP-targeted mixes, we detected heterodimers in sandwich ELISAs using either H1- and H3-specific mAbs or a broadly reactive anti-HA stem mAb in combination with the anti-A/B mAb, suggesting formation of heterodimeric vaccine proteins (Fig. 3c). When capturing with an anti-H1 mAb, dimers were detected with anti-stem and anti-H3 mAbs but not with the same anti-H1 mAb as used for capture, demonstrating formation of H1/H3 but not H1/H1 heterodimers. This result indicates a lack of heterodimers expressing two identical HA. Non-targeted heterodimers were secreted more efficiently than MHCII-targeted versions, consistent with previous observations^25,26^.

### A heterodimeric HA mix vaccine induces antibody responses against HAs included in the mix

To test the immunogenicity of the mix vaccines, we prepared mixes of 16 HA subtypes as described in Fig. 3a, but leaving out H1 (group 1) and H7 (group 2), resulting in 64 possible heterodimers. We hypothesized that a high vaccine dose might favor induction of strain-specific rather than cross-reactive antibody responses. We therefore performed an initial titration of mix vaccines which showed that vaccination with 1 µg/plasmid induced only low amounts of strain-specific antibody responses for all HA subtypes after one immunization (Supplementary Fig. 5). We therefore selected this dose for further experiments. Mice were immunized i.d. three times with either an MHCII-targeted or a non-targeted version of the DNA mix vaccine. Five weeks after the first immunization, IgG responses were observed against each of the tested HA variants included in the plasmid mix (Fig. 3d). However, the antibody titers were generally lower than those obtained after a single vaccination with plasmids encoding MHCII-targeted heterodimers with bivalent HA expression (Fig. 2a). This could be due to a lack of bivalency, lower amounts of a given HA in the mix, or both. After two boosts, antibody levels increased and reached titers of approximately 10^4^ (Fig. 3d). No differences between the MHCII-targeted and non-targeted mixes were observed. This result is consistent with the observation that the APC-targeting effect observed with bivalent antigen expression^25–29^ is less evident when antigen is monovalent^23^. Similar results were obtained after intramuscular (i.m.)/EP immunizations (Supplementary Fig. 6). Note that the HA-reactive antibodies detected in these experiments could be either cross-reactive or subtype-specific.

### Vaccination with DNA plasmid and protein mixes induces antibodies against HA subtypes not included in the mix

**Fig. 4:**
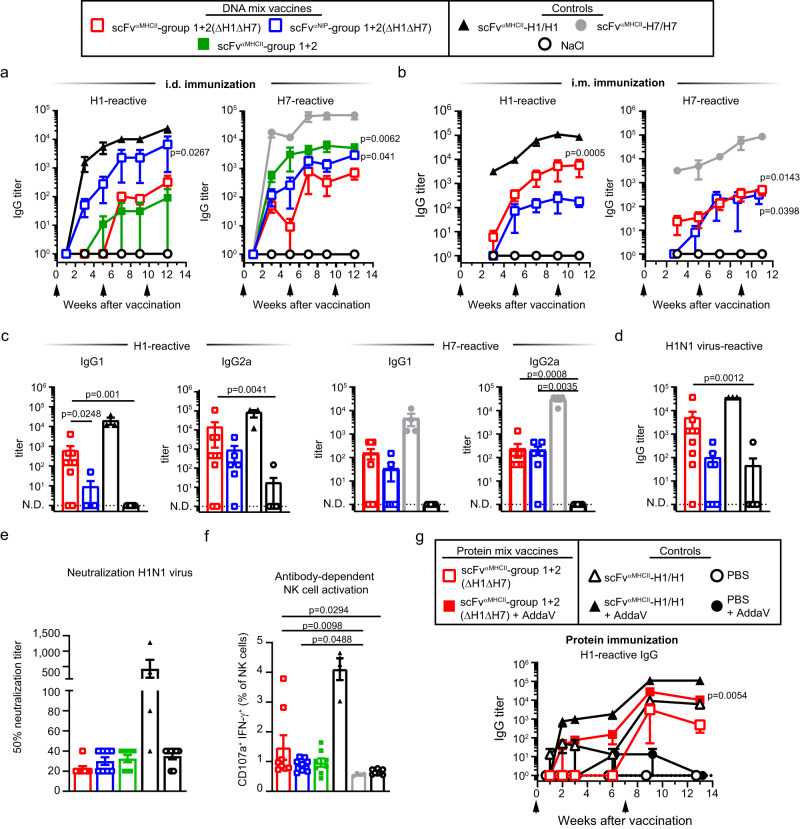
Vaccination with an HA plasmid mix induces antibodies against HA subtypes not included in the mix. **a** Female BALB/cAnNRj mice were immunized three times (arrows) intradermally (i.d.)/EP with indicated plasmid mixes (1 µg DNA/plasmid) that encoded either 16 (H1 and H7 excluded, *n* = 6) or 18 (*n* = 5) HA subtypes in either APC-targeted (scFv^αMHCII^) or non-targeted (scFv^αNIP^) formats. Positive controls were scFv^αMHCII^-targeted H1/H1 and H7/H7 bivalent vaccines (5 µg/plasmid, *n* = 4/group). Shown are H1- and H7-reactive serum IgG titers at indicated timepoints. **b** Mice were immunized three times (arrows) intramuscularly (i.m.)/EP with indicated mixes (1 µg DNA/plasmid, *n* = 7 [scFv^αNIP^-group1 + 2(ΔH1ΔH7)] or *n* = 9 [scFv^αMHCII^-group1 + 2(ΔH1ΔH7)]) or with scFv^αMHCII^-H1/H1 or scFv^αMHCII^-H7/H7 (5 µg/plasmid, *n* = 3/group) or NaCl (*n* = 9) controls. Shown are H1- or H7-reactive serum IgG titers at indicated timepoints. **c**, **d** Mice were vaccinated as in (**b**) with the indicated mix vaccines (*n* = 6 [scFv^αNIP^-group1 + 2(ΔH1ΔH7)] or *n* = 9 [scFv^αMHCII^-group1 + 2(ΔH1ΔH7)]), or with scFv^αMHCII^-H1/H1 (*n* = 3), scFv^αMHCII^-H7/H7 (*n* = 4) or NaCl (*n* = 10) controls. Shown are H1- and H7-reactive serum IgG1 and IgG2a (**c**) and IgG binding to inactivated H1N1 virus as ELSIA coat antigen (**d**) at week 11. **e**–**f** Mice were vaccinated as in (**b**) (*n* = 8 [mix vaccines and NaCl] or *n* = 4 [bivalent H1/H1 or H7/H7 control]). In vitro neutralization of PR8 virus infection of MDCK cells by week 11 sera was measured. Shown are 50% neutralizing serum titers (**e**). H1^PR8^-specific ADCC by week 11 sera was determined by measuring in vitro activation of mouse NK cells (**f**). **g** Mice were immunized twice (arrows) with purified scFv^αMHCII^-targeted ΔH1ΔH7 mix protein vaccine or scFv^αMHCII^-H1/H1 protein vaccine as control (2.5 µg protein with/without AddaVax adjuvant, *n* = 4/group). Shown is H1-reactive total serum IgG at indicated timepoints. Mean ± SEM (**a**, **b**, and **g**) or titers/percentages for individual mice plus mean ± SEM (**c**–**f**) are indicated. Responses of mix vaccines (calculated for final timepoints in (**a**), (**b**), and (**g**)) were statistically compared to NaCl or PBS with adjuvant. In **c** and **d**, responses elicited by targeted and non-targeted vaccines were also compared. Kruskal-Wallis with Dunn’s multiple comparisons test. N.D. = non-detectable. n = 1 independent experiment, except one of three for (b). Source Data file is provided.

To test whether the mix vaccine induces cross-reactive antibodies, mice were vaccinated i.d. with DNA mix vaccines that contained either 16 HAs (ΔH1ΔH7) or all 18 subtypes. IgG titers against H1 and H7 were low after a single immunization but increased after two boosts (Fig. 4a). Interestingly, the inclusion of H1 and H7 in the mix did not improve antibody responses against H1 and H7. This is to be expected since a strong anti-H1 response requires bivalency^23^. MHCII-targeting did not significantly enhance the levels of H1- and H7-reactive IgG.

We obtained similar results after i.m. immunization (Fig. 4b). Here, MHCII-targeting slightly enhanced antibody responses against H1 but not H7 (Fig. 4b–d). The cross-reactive antibodies were of both IgG1 and IgG2a subclasses (Fig. 4c). Plasmid mix vaccines also induced antibodies reactive to HA of another H1 strain, A/California/07/2009 (Cal07) (Supplementary Fig. 7). The H1-reactive antibodies bound inactivated H1N1 PR8 virus in ELISA, indicating that the epitopes bound by cross-reactive antibodies are expressed on viral particles (Fig. 4d). These results were essentially confirmed in experiments using separate mixes for group 1 and group 2 HA (Supplementary Fig. 8). Serum antibodies induced by the mix showed no detectable virus neutralization activity against PR8 H1N1 virus (Fig. 4e and Supplementary Fig. 9a), nor against Cal07 H1N1, H3N2 (A/HongKong/1/1968) or H7N1 (A/turkey/Italy/3889/1999) virus (Supplementary Fig. 9a). To explore the ability of vaccine-induced antibodies to engage in Fc-mediated antibody-dependent cellular cytotoxicity (ADCC), we tested whether induced H1-reactive antibodies could activate mouse NK cells. Mice immunized with the MHCII-targeted (ΔH1ΔH7) mix vaccine induced H1-reactive serum antibodies that enhanced levels of IFN-γ^+^ and CD107a^+^ NK cells compared to the NaCl and H7/H7 control-vaccinated mice (Fig. 4f and Supplementary Fig. 9b, c). Taken together, immunizations with plasmid mixes encoding most HA subtypes, monovalently expressed in heterodimeric vaccine proteins, induce cross-reactive antibodies against non-included HA.

The induction of cross-reactive antibodies using MHCII-targeted plasmid mixes was extended to use of a mix of purified heterodimeric proteins (Supplementary Fig. 10a, b). The heterodimeric mix of 16 HA in combination with AddaVax adjuvant induced IgG antibodies against non-included H1 (Fig. 4g). The H1-reactive antibodies were of both IgG1 and IgG2a subtypes (Supplementary Fig. 10c). In the absence of adjuvant, the MHCII-targeted mix proteins induced some anti-H1 IgG1, but not IgG2a. This result supports the idea that immunization with a plasmid mix vaccine induces transfected cells to secrete a protein mix vaccine that elicits antibody responses.

### Mix plasmid vaccines induce antibodies that cross-react between closely related HA

**Fig. 5:**
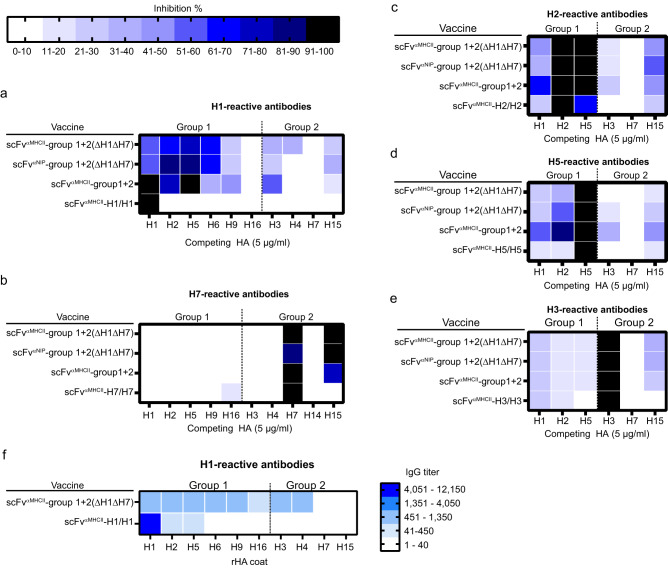
Vaccination with HA plasmid mixes induces serum antibodies that cross-react between HA subtypes. **a**, **f** Female BALB/cAnNRj mice were vaccinated thrice (week 0, 5 and 9) i.m./EP with plasmid mixes encoding the indicated mix vaccines (1 µg DNA/plasmid, *n* = 8/group). Positive controls were vaccinated with either bivalent H1/H1, H7/H7, H2/H2, H3/H3, or H5/H5, all scFv^αMHCII^-targeted (5 µg DNA/plasmid, *n* = 4/group). Sera were harvested two weeks after the final vaccination and pooled for each experimental group. **a**–**e** Pooled sera were pre-incubated with the indicated soluble competing HA proteins (5 µg/ml, *x*-axis) prior to testing for binding to coat HAs in ELISA. Heatmaps show mean % inhibition of technical triplicates. **f** H1-reactive antibodies were affinity-purified on H1PR8-conjugated Sepharose. Purified antibodies were analyzed for binding to the indicated recombinant HA proteins in ELISA. Heatmap shows mean IgG titer of technical triplicates. Each panel presents data derived from *n* = 1 independent experiment. Source data are provided as a Source Data file.

To directly analyze HA cross-reactivity of antibodies induced by the ΔH1ΔH7 16 HA plasmid mix, we performed inhibition ELISAs. Sera of vaccinated mice were pre-incubated with various recombinant HAs before adding the samples to wells coated with H1 or H7 (Fig. 5a, b). In such inhibition assays, a reduced signal in the presence of a competitor indicates that single antibody molecules bind both the coat HA and the competing HA. Binding to H1 was inhibited more effectively by group 1 HAs as compared to group 2 HAs. Moreover, the level of inhibition was most pronounced for group 1 HAs with the highest sequence homology to H1 (H2, H5, H6) (Fig. 5a, Supplementary Fig. 11). In contrast, in bivalent H1/H1-vaccinated mice, serum antibodies binding to H1 coat could only be inhibited by soluble H1 and no other HAs. (Fig. 5a). The inhibition pattern was not influenced by the inclusion of H1 and H7, nor by APC-targeting versus non-targeting.

Using H7 as a coat antigen, serum antibodies from mix-vaccinated mice were only inhibited from binding to H7 coat by H15 (~78% homology), but not by H3, H4 or H14 (each ~50% homology) (Fig. 5b, Supplementary Fig. 11). Antibodies induced by a bivalent H7/H7 vaccine had a different specificity since binding to H7 coat was only inhibited by H7 and not H15. Again, the inclusion of H1 and H7 in the mix, or APC-targeting versus non-targeting, did not influence induction of cross-reactive antibodies binding H7.

We extended the studies to HAs included in the mixes. Results with two group 1 HAs, H2 and H5, were similar to that described above for H1 (Fig. 5c, d) since HAs of closest homology inhibited the best, and APC-targeting or not did not influence the results. Using group 2 H3 as coat, we only observed effective inhibition with H3 and only weak inhibition with the other HAs (Fig. 5e).

To confirm the results of the inhibition ELISA, we used H1^PR8^ Sepharose beads to purify H1-reactive antibodies from pooled sera of vaccinated mice. Purified H1-reactive antibodies from mix-vaccinated mice [scFv^aMHCII^ group 1 + 2(∆H1, ∆H7)] bound H1 and a range of other HA subtypes in ELISA (Fig. 5f, Supplementary Fig. 12). The binding reactivity of these antibodies mimicked the cross-reactivity observed with the inhibition ELISA. Antibodies purified from mice vaccinated with bivalent H1/H1 showed minor cross-reactivity to closely related H2 and H5, but had no significant reactivity to the other HA subtypes tested. This result confirms that serum antibodies elicited by mix vaccination show broad reactivity against various HAs.

Collectively, these results show that the plasmid mix vaccine strategy induces antibodies that cross-react with the most similar HAs, suggesting that cross-reactive epitopes often may be limited to discrete groups of related HAs. Cross-reactivity between group 1 and 2 HAs appears lower than cross-reactivity within the groups, most likely due to the low sequence homology between HA of the two groups.

### Plasmid mix vaccines induce antibodies reacting to head and stems of HAs not included in the vaccine

**Fig. 6:**
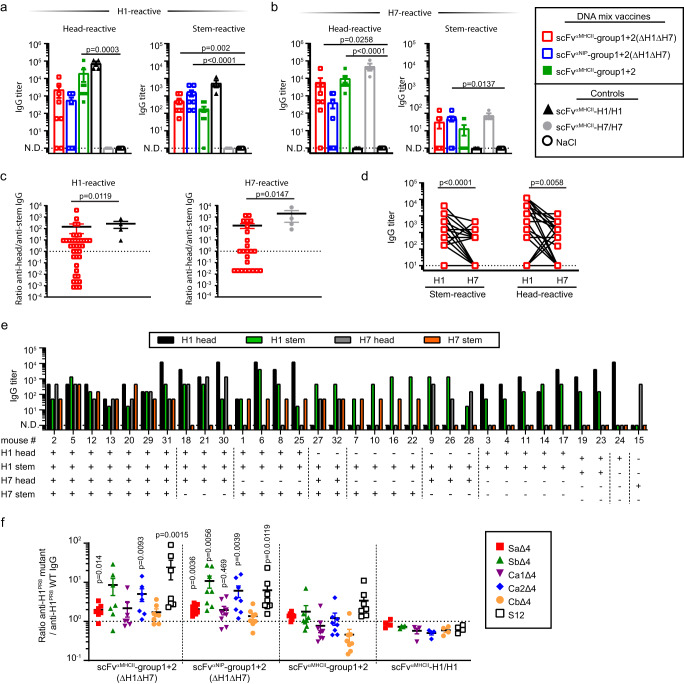
Vaccination with HA plasmid mixes induces antibodies against both the stem and head domains of HAs not included in the mix. **a**, **b** Female BALB/cAnNRj mice were immunized three times (week 0, 5 and 9) i.m./EP with indicated plasmid mixtures (1 µg DNA/plasmid, *n* = 8/group). Controls were scFv^αMHCII^-H1/H1 and scFv^αMHCII^-H7/H7 (5 µg DNA/plasmid, *n* = 4/group) or NaCl (*n* = 8). We analyzed IgG titers against the H1 (**a**) and H7 (**b**) head (left panel) and stem (right panel) domains for week 11 sera. **c**–**e** Female BALB/cAnNRj mice were vaccinated twice (week 0 and 5) with the scFv^αMHCII^-group1 + 2(ΔH1ΔH7) mix (1 µg DNA/plasmid). Serum IgG titers against the stem and head domain of H1 and H7 were analyzed four weeks after the second vaccination. **c** Ratio of IgG titers against the HA head and HA stem domain of H1 (left, *n* = 39) or H7 (right, *n* = 32) for individual mice. **d** IgG titers against stem (left) or head (right) domains of H1 (*n* = 32) and H7 (*n* = 32). Sera from individual mice, interconnected by lines. **e** IgG titers of individual mice (*n* = 32) against the H1 head (black bars), H1 stem (green), H7 head (gray) and H7 stem (orange). Mice were grouped (vertical dashed lines) according to their serum specificity. **f** Mice were vaccinated thrice as in (**a**, **b**) with the vaccines indicated on the *x*-axis (*n* = 7 [scFv^αMHCII^-group1 + 2(ΔH1ΔH7)], *n* = 8 [scFv^αNIP^-group1 + 2(ΔH1ΔH7) and scFv^αMHCII^-group1 + 2], or *n* = 4 [scFv^αMHCII^-H1/H1]). Two weeks after the third vaccination, individual sera were analyzed for IgG binding to recombinant H1^PR8^ and various mutant H1^PR8^ lacking defined head determinants (box) (Supplementary Fig. 14). The ratio of binding (AUC) to each mutant H1/H1^PR8^ WT was calculated. Shown are titers or ratios for individual mice and mean ± SEM (**a**–**c**, **f**) or titers for individual mice (**d**, **e**). Statistics were calculated using Kruskal–Wallis with Dunn’s multiple comparisons test (**a**, **b**), two-tailed Mann–Whitney (**c**) or two-tailed Wilcoxon matched-pairs signed rank test (**d**). Significant differences for ratios of serum from mix vaccinated mice compared to ratios for serum from H1/H1 vaccinated mice are indicated in (**f**). N.D. = non-detectable. *n* = 1 independent experiment. Source data are provided as a Source Data file.

The membrane proximal stem of HA is conserved and subdominant while the distal globular head is variable and immunodominant (Supplementary Fig. 1)^4,30^. We measured antibodies against the H1 and H7 globular heads and stem domains in ELISAs. We observed that all mice immunized with the group 1 + 2 (ΔH1ΔH7) 16 mix vaccine, whether MHCII-targeted or not, generated IgG that bound the H1 stem (Fig. 6a). IgG responses against the H7 stem were detected only in some mice (Fig. 6b), and titers were lower. Antibodies against globular heads of H1 and H7 were found in many but not all mice. Inclusion of H1 and H7 in the mix vaccine, containing all 18 HA subtypes, gave similar results.

These results were confirmed in a second experiment employing a large number of ΔH1ΔH7 mix-immunized mice (Supplementary Fig. 13a, b). Both H1- and H7-reactive IgG in serum from group 1 + 2 (ΔH1ΔH7)-vaccinated mice were skewed towards stem reactivity, as compared to control mice immunized with H1/H1 and H7/H7 bivalent vaccines (Fig. 6c). However, there was considerable variability in antibody specificity for H1 and H7 stem and head domains between individual mix-vaccinated mice (Fig. 6d, e), suggesting an oligoclonality of cross-reactive antibodies and a stochastic influence on specificity patterns.

### Plasmid mix vaccines induce antibodies skewed away from immunodominant antigenic sites

We further analyzed the specificity of serum from vaccinated mice to the five main immunodominant antigenic sites (Sa, Sb, Ca1, Ca2 and Cb) in the head domain of H1^4,31,32^. We coated ELISA wells with recombinant HA proteins derived from various mutated PR8 viruses in which four out of five (Δ4) antigenic sites were altered, leaving only the indicated immunodominant antigenic site intact (e.g. Sa in Sa∆4)^33^. A sixth mutant, S12, contained modifications in all five immunodominant sites^34^. The reactivity of serum from ΔH1ΔH7 mix-vaccinated mice to PR8 HA was not hampered by mutations that abrogated the immunodominant antigenic sites and showed strong reactivity to S12, indicating targeting of non-canonical antigenic sites. In contrast, sera from mice vaccinated with H1/H1 bivalent vaccines had reduced reactivity to most of the mutants, as would be expected (Fig. 6f and Supplementary Fig. 14). This indicates that the specificity pattern of antibodies elicited by mix vaccines is shifted away from the immunodominant antigenic sites. The inclusion of H1 (and H7) in the mix vaccine resulted in a reactivity pattern to the H1^PR8^ mutants that is more similar to that induced by the H1/H1 bivalent vaccine.

### Plasmid mix vaccine induces HA-specific T-cell responses

**Fig. 7:**
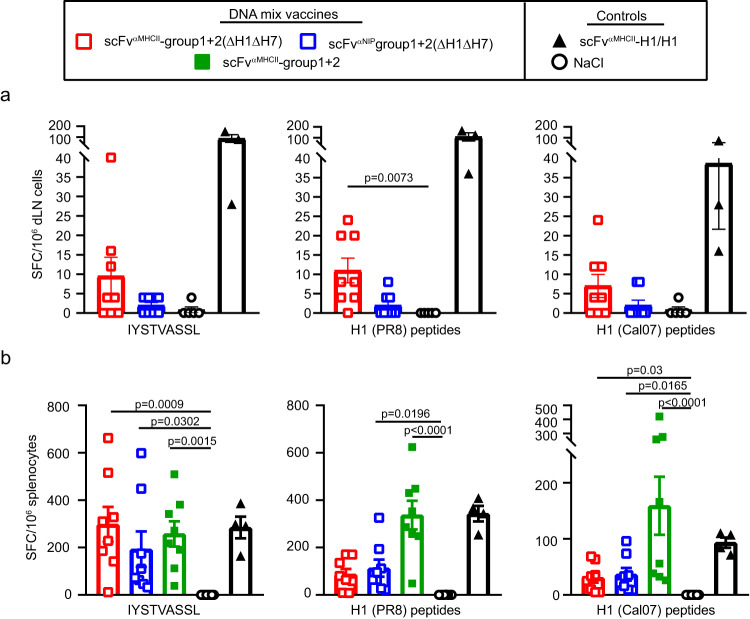
The HA mix vaccine induces IFN-γ secreting T cells. **a** Female BALB/cAnNRj mice were immunized once i.m./EP with DNA HA mix vaccines (1 µg DNA/plasmid, *n* = 8/group) as indicated. As controls, mice were vaccinated with scFv^αMHCII^-H1/H1 (H1 from PR8, 5 µg/plasmid, *n* = 3) or NaCl (*n* = 5). Draining lymph nodes (dLN) were collected 5 days after immunization, and IFN-γ secretion was measured by ELISPOT after stimulation with either IYSTVASSL (H1) peptide, or PR8- or Cal07-overlapping peptides (both H1N1). **b** Female BALB/cAnNRj mice were vaccinated three times (week 0, 5 and 9) i.m./EP with DNA HA mix vaccines (1 µg DNA/plasmid, *n* = 8/group) as indicated. Control mice were vaccinated with scFv^αMHCII^-H1/H1 (H1 from PR8, 5 µg/plasmid, *n* = 4) or NaCl (*n* = 8). Spleens were harvested two weeks after the third immunization and stimulated with HA peptides as in (**a**). IFN-γ secreting cells were analyzed by ELISPOT. Shown are the number of spot-forming cells (SFC) per 10^6^ cells for individual mice and mean ± SEM. Significance was calculated using Kruskal–Wallis with Dunn’s multiple comparisons test, comparing the mix-immunized groups with the NaCl control and scFv^αMHCII^ targeted with non-targeted control. Only differences scored as significant are indicated. *n* = 1 independent experiment. Source data are provided as a Source Data file.

To test if the mix vaccines could induce T cell responses, we immunized mice for various number of times with different plasmid mixes and analyzed T cell responses in draining lymph nodes (dLN) and spleen employing three sources of H1 peptides: IYSTVASSL, a K^d^-restricted CD8 epitope^35^, conserved between the group 1 HA subtypes (Supplementary Fig. 1); an overlapping PR8 peptide pool; and an overlapping Cal07 peptide pool (Fig. 7, Supplementary Fig. 15a–c). Based on previous experiments with APC-targeted DNA vaccines^25^, T-cell responses were detected by the production of IFN-γ. The results show that: (i) the group 1 + 2 (∆H1∆H7) mix, lacking H1, induced IFN-γ T cell responses towards all three sources of H1 peptides; (ii) weak T cell responses were detected in dLN already 5 days after a single mix immunization and increased by day 11 post-vaccination (Fig. 7a, Supplementary Fig. 15a); (iii) three mix immunizations elicited increased responses, almost equal to those elicited by a positive control vaccine (MHCII-targeted H1/H1) (Fig. 7b); (iv) MHCII-targeting of mix vaccine enhanced responses only early (day 5) after a single vaccination (Fig. 7a); (v) The 18 HA mix vaccine, where PR8 H1 was included, induced higher T cell responses against H1 peptides than did the ΔH1ΔH7 mix (Fig. 7b, Supplementary Fig. 15a, b); (*vi*) H1-specific responses were mainly seen for CD8^+^ T cells (Supplementary Fig. 15b). Taken together, mix vaccines elicit T cells cross-reactive to non-included HA (H1). Inclusion of H1 in the mix, MHCII-targeting, and multiple immunizations, enhanced responses.

### Cross-reactive antibodies mediate protection against challenge with an HA heterologous influenza virus

**Fig. 8:**
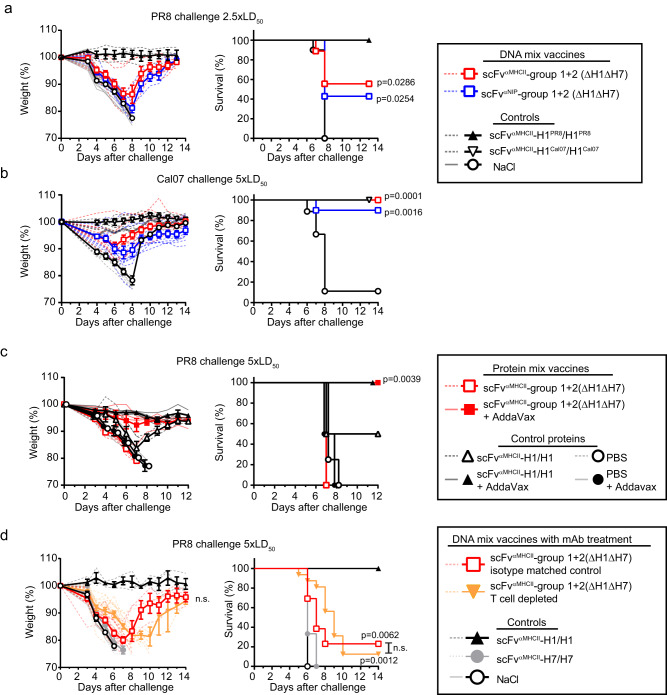
The HA mix vaccine induces partial protection against heterologous influenza virus. **a** Female BALB/cAnNRj mice were immunized three times (week 0, 5 and 9) i.m./EP with the indicated DNA HA mix vaccines (1 µg DNA/plasmid, *n* = 7 [scFv^αNIP^-group1 + 2(ΔH1ΔH7)] or *n* = 9 [scFv^αMHCII^-group 1 + 2(ΔH1ΔH7)]). As controls, mice were immunized with scFv^αMHCII^-H1/H1 (H1 from either PR8, 5 µg/plasmid, *n* = 3) or NaCl (*n* = 10). Two weeks after the third immunization, mice were infected with 2.5xLD_50_ of H1N1 PR8 virus. **b** Mice were vaccinated as in (**a**) (*n* = 6 [scFv^αMHCII^-H1Cal07/H1Cal07], *n* = 9 [NaCl] or *n* = 10 [scFv^αNIP^- and scFv^αMHCII^-targeted mix vaccines]). Two weeks after the third immunization, mice were infected with 5xLD_50_ of H1N1 Cal07 virus. **c** Female BALB/cAnNRj mice were vaccinated twice (week 0 and 6) with scFv^αMHCII^-targeted HA mix protein vaccine or bivalent H1/H1 (PR8) as control (2.5 µg protein with or without AddaVax as adjuvant, *n* = 4/group). Seven weeks after the second immunization, mice were infected with 5xLD_50_ of H1N1 PR8 virus. **d** Female BALB/cAnNRj mice were immunized twice (week 0 and 5) i.m./EP with DNA HA mix vaccines (1 µg DNA/plasmid, n = 29). Controls were vaccinated with scFv^αMHCII^-targeted H1/H1 (PR8) or H7/H7 (5 µg/plasmid, *n* = 3/group) or NaCl (*n* = 6). Four weeks after the second immunization, mice were challenged with 5xLD_50_ of H1N1 PR8. Starting two days before challenge, mice vaccinated with the scFv^αMHCII^ mix received either anti-CD4 plus anti-CD8 mAbs (*n* = 16) or isotype matched control mAbs (*n* = 13), every other day. Weight loss (mean ± SEM, left) and survival (right) are indicated in the panels. Mice were euthanized when they reached 80% of their initial weight and removed from the weight curves. Mean ± SEM is indicated with thick colored lines and symbols. Weight of individual mice indicated in stippled and faded lines. Significance was calculated using two-tailed Mantel–Cox (survival comparing mix vaccines to NaCl) or two-way ANOVA (weight curves). For T cell-depleted mice and isotype-matched controls compared in (**d**), n.s. = not significant. *n* = 1 independent experiment. Source data are provided as a Source Data file.

Next, we explored if the cross-reactive antibodies and T cells induced by the HA mix vaccines could protect against challenge with a heterologous virus that expresses an HA subtype not included in the mix. Mice were immunized thrice with a group 1 + 2 (ΔH1ΔH7) mix vaccine. Four weeks after the final vaccination, mice were challenged with a lethal dose (2.5xLD_50_) of H1 PR8 virus. Both MHCII-targeted and non-targeted mix vaccines induced a partial protection, with most mice losing a considerable amount of weight. However, both vaccines protected about 50% of the mice from reaching the endpoint of 20% weight loss (Fig. 8a). The positive control, MHCII-targeted H1/H1(PR8), induced full protection, as expected^25^. The mix vaccines reduced viral titers in the lungs of PR8-challenged mice (Supplementary Fig. 16a). When extending the challenge experiments to other H1 and H7 viruses we obtained divergent results. The plasmid mix vaccine did not offer significant protection to H7N1 virus (A/turkey/Italy/3889/1999) (Supplementary Fig. 16b) which, in our hands, is more pathogenic than PR8 virus. In contrast, mix-vaccinated mice were fully protected against challenge with 5 x LD_50_ Cal07 H1N1 virus two weeks after a third immunization (Fig. 8b). When the vaccine was generated as protein, two immunizations with the MHCII-targeted ΔH1ΔH7 mix protein fully protected mice against challenge with 5xLD_50_ PR8 virus, but only when delivered with AddaVax adjuvant (Fig. 8c).

The partial protection against the PR8 heterologous challenge could be due to H1 cross-reactive antibodies (Figs. 4–6) or T cells (Fig. 7) induced by the group 1 + 2 (ΔH1ΔH7) mix vaccination. To test the contribution of antibodies and T cells, mice vaccinated twice with the MHCII targeted group 1 + 2 (ΔH1ΔH7) mix were depleted of T cells by injection of anti-CD4 and anti-CD8 mAbs (or isotype-matched control mAbs) starting two days prior to a challenge with 5 x LD_50_ of PR8 virus (Fig. 8d, Supplementary Fig. 16c). T cell-depleted mice were as well protected as non-depleted mice. However, only 10–20% of mice survived the challenge in either group, perhaps due to the high dose of influenza virus used for challenge. Nevertheless, the protection conferred by mix-vaccination, either in the absence or presence of T cell depletion, was significant compared to negative controls (NaCl and MHCII-targeted H7/H7 immunized mice). These results support a role for cross-reactive antibodies as mediators of the protection against virus with an HA subtype that was not included in the mix vaccine.

## Discussion

Current influenza vaccines induce predominantly strain-specific antibodies. These are protective as long as the infecting influenza strain matches the vaccine strains. An important goal in influenza vaccine research is therefore to develop universal vaccines that induce broadly reactive antibodies that protect against many, if not all, influenza strains.

We recently observed that MHCII-specific heterodimeric vaccine proteins generated APC-B cell synapses^23^. Moreover, antigen bivalency, meaning that heterodimeric proteins express two identical antigens on both antigenic arms, was necessary to induce potent antibody responses, most likely due to BCR crosslinking. We here hypothesized that in a mixture of many different heterodimeric vaccine proteins, each of which always expresses two different but related antigens on its two arms, only conserved epitopes shared between the related but different antigens would be bivalent in a single heterodimeric vaccine protein. Consequently, B cells with a BCR that recognize these conserved bivalent epitopes should preferentially be stimulated. To test this hypothesis of valency-based immuno-selection, we generated a mixture of DNA plasmids that encoded heterodimeric vaccine proteins expressing 16 different IAV HA subtypes, each heterodimer carrying two different HAs. The plasmid mix induced cross-reactive antibodies against H1 and H7, which were not included in the mix. The cross-reactive antibodies partially and fully protected against two different hetero-subtypic H1 virus challenges in the absence of CD4^+^ and CD8^+^ T cells.

Analysis of sera from mix-immunized mice suggested that a fraction of induced antibodies was cross-reactive between H1 (or H7) and certain HA subtypes expressed in the mixed vaccine. These cross-reactive antibodies apparently bound shared conserved sites that ordinarily are subdominant. In inhibition ELISA, cross-reactive antibodies were only inhibited by HAs that were most homologous to non-included H1 (or H7) suggesting a restricted sharing of conserved epitopes among all HA subtypes. Supporting this notion, the mix vaccines elicited antibodies that cross-reacted between related HAs that had been included in the mix, again indicating that the vaccination strategy skews antibody responses towards conserved epitopes shared between similar HAs.

The assay used here demonstrated cross-reactivity at the polyclonal antibody level. Generating mAb from mix-vaccinated mice should enable structural insights into the localization of conserved epitopes recognized by broadly cross-reactive antibodies. Our results suggest that relevant epitopes are localized to both the head and the stem of HA. There was, however, considerable heterogeneity between individual mice, indicating that cross-reactive B cell responses may be oligoclonal.

Sera from mix-vaccinated mice did not have any meaningful neutralizing activity, but they were able to activate NK cells. Broadly reactive antibodies against HA often protect through FcγR-dependent mechanisms, such as antibody-dependent cellular cytotoxicity^36–38^. Hence, it might be important that mixed plasmid vaccines skewed cross-reactive antibody responses towards the IgG2a subclass over IgG1. Important in this respect, the specificity of targeted vaccines for surface molecules on APC influences antibody isotype. For example, chemokines MIP-1α^26^ and Xcl1^39^ predominantly induce FcγR-activating IgG2a antibodies. The MHCII-targeting used herein induced both IgG1 and IgG2a cross-reactive antibodies. Thus, substituting this targeting unit with MIP-1α or Xcl1 should further increase the IgG2a profile of cross-reactive antibodies and improve the ability of serum antibodies to engage in Fc-mediated ADCC.

Other approaches that combine different HA variants into one vaccine have been used to induce cross-reactive antibodies against HA. We previously showed that simultaneous immunization with six group 1 HA subtypes expressed in MHCII-targeted vaccine proteins induced antibodies against H1 not included in the mix^40^. However, that study used DNA plasmids encoding protein chains that could form homodimers. Therefore, a fraction of secreted dimers are likely to bivalently express the same HA^40^, making interpretation more complicated compared to the present use of exclusively heterodimeric molecules.

Kanekiyo et al. showed that heterotypic nanoparticles with eight different H1 head constructs induced broadly reactive antibodies^41^. The authors suggested that having an avidity advantage improves the activation of cross-reactive B cells, and subverts the activation of strain-specific B cells unable to bind shared epitopes^41^. Other groups have shown that intranasal delivery of a mixture of four different virus-like particles, each carrying a different HA subtype, induced significant protection against a variety of heterosubtypic IAV strains. However, this protection was likely not due to cross-reactive antibodies^42^. Sequential immunizations with chimeric HAs that combine the same stem domain with different irrelevant head domains induce cross-reactive antibody responses against the subdominant HA stem domain^43^. The computationally optimized broadly reactive antigen (COBRA) methodology, using multiple rounds of layered consensus building to generate influenza vaccine HA immunogens, induced broad hemagglutination inhibition activity as a head-based strategy^44^.

The use of “mix vaccines” has also been studied for several other infectious diseases, including malaria^45–47^, HIV^48^ and infection with SARS-like viruses^49^. In silico work suggests that combining multiple malaria antigens in one vaccine favors affinity maturation towards cross-reactive antibodies^45^. Immunization with four different variants of AMA1 malaria antigen in rabbits enhanced cross-reactive responses as compared to immunization with one single AMA1^46^. In silico models with HIV gp120 mix vaccines suggest that the generation of an optimal mix vaccine requires careful consideration of the number of antigens, their concentration, and the mutational distance between different antigens^48^. Single mosaic nanoparticles expressing the receptor binding site of eight SARS-CoV-2 related viruses induced cross-reactive antibodies targeting more conserved sites in mice and non-human primates^49^.

The vaccination strategy used herein may be optimized and expanded. Many more HAs may be introduced into an expanded plasmid mix. Alternatively, mixes with only variants of H1, H3 or IBV HA could be generated to induce broad protection against strains that currently cause seasonal influenza epidemics. Besides influenza, the mixed strategy could be valuable for generating vaccines against other viral diseases, such as the highly polymorphic gp120 of HIV viruses or the spike of SARS-CoV-2.

The mixed vaccine could be delivered both as plasmids and proteins, with similar results. This is to be expected since cells transfected with many (or all) mixed plasmids are likely to secrete heterodimeric molecules assembled stochastically in the ER^22,23^. Most likely, the heterodimeric mix vaccine could also be delivered as modified mRNA. Supporting this possibility, a recent publication from Arevalo et al. describes a multivalent mRNA vaccine encoding 18 IAV and 2 IBV HA subtypes. Vaccinated mice developed antibodies against all the included HA subtypes. However, it is unclear to what extent antibodies were cross-reactive or not^50^.

Based on the present results using DNA delivery, and the apparently high immunogenicity of mRNA vaccines^50^, one may ask whether the valency-based immuno-selection mechanism afforded by our mix APC-targeting heterodimers would be maintained upon mRNA rather than DNA delivery. If so, the high immunogenicity of mRNA vaccines could nevertheless be outweighed by several advantages of plasmid delivery such as (i) ease of engineering and production, (ii) independence of a cold chain, and (iii) needle-free jet delivery^51^.

A vaccine that reliably induces protection against multiple influenza strains may eliminate the need for annual updates of seasonal influenza vaccines. An important question is when such a vaccine should be administered since previous exposures to influenza viruses could thwart its effectiveness due to imprinting (original antigenic sin)^52–55^. Perhaps a valency-based mix vaccine should be the first immunization to influenza, given very early in childhood, before any imprinting has occurred. Arguing against a necessity of early mix vaccination, Arevalo et al. showed that prior infection of mice with an H1N1 virus did not hamper the induction of antibodies elicited by their mRNA mix vaccine^50^.

## Methods

### Mice, cell lines, and viruses

Female BALB/cAnNRj/c mice (Janvier Labs, Le Genest Saint Isle, France or Taconic biosciences inc, NY, USA), 6-8 weeks old, were used for the experiments. All experiments were reviewed and approved by the Norwegian Animal Research Authority and were carried out in accordance with the recommendations from the Guide for the Care and Use of Laboratory Animals of the Norwegian National Institute of Health. HEK293E cells were from ATCC (RRID:CVCL0045, cat# CRL-1573, Manassas, USA). Madin-Darby canine kidney (MDCK) cells were likewise purchased from ATCC (RRID:CVCL0422, cat# CCL-34). Cells lines were cultured in complete medium: RPMI 1640 (R2405, Invitrogen) with 10% heat-inactivated FCS (F7524, Sigma), 24 mg/l gentamicin (Gensumycin®, VNR 453130, Sanofi-Aventis Norge AS), 50 µM monothioglycerol (M6145, Sigma), 1 mM sodium pyruvate (11360-039, Gibco), and 0.1 mM non-essential amino acids (11140-035, Gibco). The growth medium for MDCK cells contained the same supplements, but in DMEM (41965062, Life Technologies). Mouse-adapted (VR-95) and MDCK-adapted (VR-1469) viruses from strain A/Puerto Rico/8/1934 (H1N1, PR8) were purchased from ATCC. Virus strain A/California/07/2009 (H1N1, Cal07) was kindly donated by Dr. A. Germundsson (The National Veterinary Institute, Oslo, Norway), the LD_50_ dose was established by the Reed and Muench method and titrations of virus in mice^25^. Virus from strain A/turkey/Italy/3889/1999 (H7N1) was kindly donated by Prof. Rebecca Cox (UiB, Bergen, Norway), and was mouse adapted in our lab before use.

### Generation of phylogenetic tree and multiple sequence alignment of HA proteins

The phylogenetic tree was generated using a Clustal Omega multiple alignment of the amino acid sequences of the HA proteins used in this paper. The tree was made with the neighbor-joining method without distance correction (URL: https://www.ebi.ac.uk/Tools/msa/clustalo/^56^). The radial tree was visualized using an online version of TreeDyn 198.3 (URL: http://www.phylogeny.fr/^57^). The graphic amino acid alignment of the HA proteins used in this paper was made using blastp suite (URL: https://blast.ncbi.nlm.nih.gov/Blast.cgi?PROGRAM=blastp&PAGE_TYPE=BlastSearch&LINK_LOC=blasthome^58^).

### Cloning of heterodimeric vaccine vectors

DNA vaccines were encoded in the pLNOH2 vector under a CMV promotor and the signal peptide sequence from the V_H_ gene of B1-8 mAb^59^. The targeting units, anti-MHCII (I-E^d^, scFv^αMHCII^ from mouse 14-4-4 S mAb) and anti-NIP (scFv^αNIP^ from mouse B1-8 mAb) were subcloned from previously constructed plasmids^27^. The heterodimerization unit was based on a modified Fos-Jun based leucine zipper motif consisting of ACID (QLEKELQALEKENAQLEKELQALEKELAQ) and BASE (QLKKKLQALKKKNAQLKKKLQALKKKLAQ) sequences on separate protein chains. Sequences for the A/B dimerization motif were kindly donated by Dr. Elisabeth Mellins^24^. Within the previously constructed vaccine plasmids, two A or two B motifs were spaced by a linker (GGGSGGLTKFGGSTTAPS), and placed C-terminal of the hinge exon 1 from human IgG3^22^. The HA subtype strains used for H1-18 of IAV are listed in Table 1, and the amino acid sequences of each HA subtype strain are shown in Supplementary Fig. 1. H1 from PR8 and A/California/07/2009 (Cal07)^25^, H5, H6, H8, H9, H11, H13^40^ and H7^60^ were subcloned from existing plasmids with restriction enzyme SfiI^25^. The remainder of the HA genes were amplified from mammalian codon optimized cDNA (Sino Biological, category numbers indicated in Supplementary Table I) by PCR with specifically designed primers (Sigma):

H2_16_5′: ACC*GGCCTCGGTGGCCTG*GACCAGATTTGTATTGGCTACC,

H2_539_3′: ACC*GGCCCTGCAGGCC***TCA**GAGGCTCAGGGATCCAG,

H3_17_5′: *GGCCTCGGTGGCCTG*CAAGACCTTC,

H3_538_3′: A*CCGGCCCTGCAGGCC***TCA**TCAAATAGCAAAAG,

H4_17_5′: ACC*GGCCTCGGTGGCCTG*CAGAACTACACAGGCAACC_,_

H4_537_3′: ACC*GGCCCTGCAGGCC***TCA**GGAGATGCTGAAGGAGAAC,

H10_18_/H15_19_5′: ACC*GGCCTCGGTGGCCTG*GACAAGATTTGTCTGGGACACC,

H10_534_/H15_543_3′: ACC*GGCCCTGCAGGCC***TCA**GGAGGCTCCAAAGGAGAAC,

H12_18_5′: ACC*GGCCTCGGTGGCCTG*GACAAGATTTGTATTGGCTACCAG,

H12_239_3′: ACC*GGCCCTGCAGGCC***TCA**CAGGGAGGATGCCACAGA,

H14_18_5′: ACC*GGCCTCGGTGGCCTG*CAGATTACCAATGGCACCAC,

H14_541_3′: ACC*GGCCCTGCAGGCC***TCA**ACTCATACTGAAGGAAATCCACAG,

H16_20_5: ACC*GGCCTCGGTGGCCTG*GACAAGATTTGTATTGGCTACCTG,

H16_541_3′: ACCGGCCCTGCAGGCC**TCA**CAGCACAATGCTGGAGGC,

H17_19_5′: ACC*GGCCTCGGTGGCCTG*GACAGGATTTGTATTGGCTACCAG,

H17_541_3′: ACCGGCCCTGCAGGCC**TCA**CAGCACCACAGAGGAGGC,

H18_15_5′: ACC*GGCCTCGGTGGCCTG*GACCAGATTTGCATCGGC, and

H18_538_3′: ACC*GGCCCTGCAGGCC***TCA**CAGCACGACACTTGAGGC (SfiI sites indicated in italic, stop codon in bold). Primers were designed to exclude the intrinsic HA signal peptide and most of the transmembrane region from the HA gene. The primer names show the amino acid numbers included for the individual HA subtypes.

### Transient transfection of HEK293E cells

HEK293E cells (5 × 10^5^) in 5 ml complete medium per well were seeded in 6 well culture plates (3506, Corning), and incubated at 37 °C with 5% CO_2_. The following day, the complete medium was replaced by FreeStyle™ 293 serum free medium (12338002, Life Technologies) with 24 mg/l gentamicin before cells were transfected with 2.5 µg DNA of A and B plasmids (5 µg in total) with 10 µg Polyethylenimine (23966, Polysciences). To produce HA mix proteins with 18 different plasmids, 5 µg DNA/plasmid and 200 µl polyethylenimine were used for transient transfection of HEK293E cells in T175 flasks. Supernatants were collected after 3-4 days, and centrifuged 5 min at 350 xg. For Western blot analysis and HA mix heterodimer ELISA, supernatants were concentrated 10 times using Vivaspin 20 spin columns with 10 kDa cut off (VS2002, Sartorius).

### Protein purification

For the production of larger amounts of MHCII-targeted ΔH1ΔH7 mix and MHCII-targeted H1/H1 vaccine proteins, HEK293E cells were transfected with a total of 250 µg DNA in BD Falcon® 5-layer multiflasks (353114, Corning) in FreeStyle^TM^ 293 serum free medium. Proteins were purified using an affinity column with anti-A/B mAb (clone 2H11, produced from hybridoma kindly provided by E.L. Reinherz^61^) coupled to CNBr activated Sepharose 4 Fast Flow (17-0981-01, GE healthcare).

### ELISA to detect vaccine proteins

High binding 96 well plates (3590, Corning) were coated with 1 µg/ml anti-A/B mAb, or anti-IAV H1N1 (A/Puerto Rico/8/1934) HA (clone H36-4-52, produced from hybridoma provided by Siegfried Weiss^62^ mAb in PBS, incubated at 4 °C overnight, and blocked with 200 µl blocking buffer (1% w/v BSA in PBS with 0.02% w/v Na-Azide) per well. Supernatants from transfected HEK293E cells were added (50 µl/well) and serially diluted 3-fold in triplicates with ELISA buffer (0.1% w/v BSA and 0.2% Tween 20 in PBS/Na-Azide). Plates were incubated for 2 hours at room temperature. Vaccine proteins were detected for 1 hour with 1 µg/ml biotinylated anti-H1 mAb, a cross-reactive rabbit mAb specific for IAV HA stem (1:3000, clone #2, 86001-RM01, Sino Biological), or 1 µg/ml rabbit-anti-AIV H3N2 (A/Brisbane/10/2007) HA mAb (clone# 104, 11056-R104, Sino Biological). Biotinylated mAb was detected with streptavidin-Alkaline Phosphatase (ALP) conjugate (1:3000, RPN1234, Southern Biotech) and rabbit mAbs with polyclonal (p) anti-rabbit IgG Ab-ALP conjugate (1:3000, A3687, Sigma Aldrich). Signals were developed with phosphatase substrate (1 mg/ml, P4744, Sigma) dissolved in diethanolamine substrate buffer. OD_405nm_ was measured using EnVision 2104 Multilabel Reader with EnVision Manager 1.12 software (PerkinElmer).

### Western blot analysis

Concentrated supernatants were normalized on a dot blot with 3-fold serial dilutions of supernatant in PBS and detected with 0.33 µg/ml anti-A/B mAb. Approximately similar amounts of vaccine proteins were run on a Bolt™ 4-12% Bis-Tris Plus gel (NW04122BOX, Invitrogen). Proteins were transferred to a PVDF membrane using the iBlot2 gel transfer system with a PVDF transfer stack (IB24002, Invitrogen). Membranes were blocked using 2% blocking reagent (RPN2125, GE Healthcare) in 0.1% tween 20 in PBS. Vaccine proteins were detected with 0.33 µg/ml biotinylated anti-A/B mAb followed by streptavidin-HRP conjugate (1:3000, 7100-05, Southern Biotech). Blots were captured using G:box Chemi XX6 device (SYNGENE).

### DNA vaccination

Mice were anaesthetized by an interperitoneal injection with 0.06 ml/10 gr body weight of a cocktail of Tiletamin/Zolazepam (250 mg/ml, Zoletil Forte, Vibrac), xylazine (Rompun, 20 mg/ml, Bayer Animal Health), and fentanyl (50 µg/ml, Actavis). Plasmids were purified using Endofree Megaprep plasmid purification kits (12381, Qiagen), and recovered in 0.9% NaCl solution (VNR 451773, B. Braun). Bivalent scFv^αMHCII^-H1-18/H1-18 vaccines contained 0.05 µg/µl − 0.5 µg/µl of both A and B DNA plasmids (0.1–1 µg/µl DNA in total). Mix vaccines contained 0.02 µg/µl in 50 µl (i.d. vaccination) or 0.01 µg/µl in 100 µl (i.m. vaccination) of each included plasmid vector (for a final dose of 1 µg/plasmid in total per mouse). For i.d vaccinations, 25 µl DNA was injected in the lower back near the base of the tail on both flanks, immediately followed by EP with DermaVax (BTX Harvard Apparatus), delivered in two pulses of 450 V/cm × 2.5 µs plus eight pulses of 110 V/cm × 8.1 ms. For i.m. vaccination, 50 µl DNA was injected in the *quadriceps femoris* muscle of each leg, followed by EP with Elgen 1000 Needle Electroporator (Inovio Biomedical), delivered as 5 × 60 ms at 50 V/400 mA with 200 ms delay. In many experiments, mice were immunized three times, at week 0, 5 and 9 unless otherwise specified. Blood samples were collected from the saphenous vein, spun down twice, and sera frozen at −20 °C. Blood was collected at weeks 1, 3, on the day of immunization and two weeks after the second and third immunization.

### Protein vaccination

Proteins (50 µg/ml in PBS) were combined in a 1:1 ratio with adjuvant AddaVax (vac-adx-10, Invivogen) or PBS for non-adjuvanted controls. Hind legs of mice were shaved, and 50 µl vaccine protein was injected in the *quadriceps femoris* muscle on each side, resulting in a final vaccine dose of 2.5 µg protein per mouse. Mice were immunized with protein twice, at week 0 and 7, and blood was collected at 1, 2, 3, 6, 9 and 13 weeks after the first immunization.

### ELISA for HA-reactive antibodies in serum

The serum ELISAs were run as described above for protein ELISAs except for the following: Recombinant HA or HA1 subunit protein (0.5 µg/ml, Sino Biologicals, category numbers indicated in Supplementary Table I), or inactivated mouse adapted H1N1 PR8 virus (1:1600, 10100782, Charles River Laboratories) in PBS were used as coat. Serum was serially diluted 3-fold in ELISA buffer starting at 1:50. HA-reactive antibodies were detected with anti-mouse IgG pAb-ALP conjugate (1:5000, A2429, Sigma Aldrich). IgG subtypes were detected with biotinylated 1 µg/ml anti-mouse IgG1^[a]^ (1:500, clone10.9, 553500, BD Biosciences) or anti-mouse IgG2a^[a]^ (1:500, clone 8.3, 553502, Biosciences) followed by detection with streptavidin-HRP (1:5000, Southern Biotech) and 100 µl TMB solution (CL07-1000ML, Merck Millipore). Signal development was stopped with 100 µl 1 M sulfuric acid, and OD_450nm_ was measured. The Ab titer was defined as the final dilution that resulted in OD_405nm_ or OD_450nm_ values > (2 x mean) of NaCl-vaccinated mice. Samples with a titer <50 were given an endpoint titer of 1.

Some ELISAs were performed with recombinant HA protein from PR8 viruses that were mutated to abolish four out of five of the major H1 antigenic sites (Δ4 mutants)^33^, and a mutant that was altered all five major antigenic sites, abrogating binding of most tested mAb towards these sites (S12)^34^. The mutant proteins were produced and purified as described previously^33^. The recombinant HA proteins were used as ELISA coat at a concentration of 1 µg/ml. Serum IgG titers towards the mutated HA was analyzed as described above.

### ELISA for detection of HA stem-specific antibodies

HEK293E cells were transfected with plasmids encoding dimeric fusion proteins that expressed a scFv against the hapten 4-ethoxymethylene-2-phenyl-2-oxazoline-5-one (phOx)^63^ linked to the human Ig-hinge regions h1 and h3, and CH3 from human IgG3 as dimerization unit^27^, and to the HA stem derived from either H1 or H7 as antigenic unit^64^ to form scFv^α^phOx-CH3-HA stem proteins. phOx (E0753-1G, Sigma) was coupled to BSA. The resulting phOx-BSA (1 µg/ml) was used as coat, and supernatant with scFv^αphOx^-C_H_3-HA stem proteins was added and incubated for 2 hours. The scFv^αphOx^ -C_H_3-HA stem supernatant was exchanged with fresh supernatant twice to obtain a saturated coating with protein dimers. The presence of scFv^αphOx^ -C_H_3-HA stem in the ELISA was confirmed through binding of mAb towards the human IgG3-based dimerization motif (anti-human IgG-Biotin, 1 µg/ml, clone HP-6017, B3773, Sigma) and rabbit anti-HA stem mAb (1:3000) detected with anti-rabbit IgG Ab-ALP. Mice sera were serially diluted 3-fold in ELISA buffer, starting at 3:50. HA stem-specific antibodies were detected with anti-mouse IgG-ALP conjugate.

### Microneutralization assay

The viral 50% TCID of MDCK-adapted H1N1 PR8 influenza virus was determined using the Reed-Muench method^65^. Serum from vaccinated mice was mixed in 1:3 ratio with receptor destroying enzyme from cholera filtrate (C8772, Sigma) incubated at 37 °C for 20 hours, and heat-inactivated at 56 °C for 60 min. Starting at 1:10 dilution, 2-fold serial dilutions of treated serum was incubated with equal volumes of 100xTCID_50_ PR8 virus in virus diluent (DMEM supplemented with 48 mg/l gentamicin, 100 μM monothioglycerol, 2 mM sodium pyruvate, 0.2 mM non-essential amino acids, 1% bovine albumin fraction V (10735078001, Roche), 0.02 M HEPES (83264-100ML-F, Sigma), and 1 µg/ml TPCK-Trypsin (T1426-100MG, Sigma)) for 2 hours on 96-wells plates at 37 °C at 5% CO_2_. As controls, four wells on each plate contained only virus and four wells contained only virus diluent. MDCK cells (2 × 10^2^) were added to each well and incubated for 18 hours at 37 °C and 5% CO_2._ Cells were washed with PBS, fixed with cold 80% acetone for 10 min, and viral nucleoprotein was detected in ELISA using biotinylated anti-nucleoprotein mAb (1 µg/ml, clone HB65, produced from hybridoma, H16-L10-4R5, ATCC) followed by streptavidin-ALP (1:3000). Plates were developed and read as described above.

### Analysis of antibody-mediated NK cell activation

To determine ADCC, we adapted a protocol from Jegaskanda et al.^36^ Briefly, spleens were harvested from naïve BALB/cAnNRj/c mice and dissociated in gentleMACS™ C Tubes (130-096-334, Miltenyi Biotech). Splenocytes were treated with Tris-buffered ammonium chloride for 5 min on ice. Cells were filtered through a 70 nm Nylon strainer (732-2758, VWR). NK cells were isolated using an NK cell negative selection kit (130-115-818, Miltenyi Biotech) according to the manufacturer’s protocol. NK cell purity was determined by Flow cytometry. Purified NK cells were pre-activated overnight with IL15 (10 ng/ml, 447-ML-010, R&D systems) in a complete medium at 37 °C and 5% CO_2_. High-binding 96 well plates were coated with 0.25 µg/ml recombinant H1^PR8^ protein in PBS, at 4 °C overnight. Coated plates were washed five times with PBS and blocked for 2 hours in 200 µl 1% w/v BSA in PBS at room temperature. Then, wells were incubated with heat-inactivated serum from vaccinated mice at 4 °C overnight. The next day, plates were washed five times with PBS, and 0.5 × 10^6^ pre-activated NK cells resuspended in 100 µl complete cell medium supplemented with 10 ng/ml IL15, anti-mouse CD107a (LAMP-1)-PE antibody (2 µg/ml, clone 1D4B, 121612, Biolegend) and GolgiPlug™ protein transport inhibitor (1:1000, 555029, BD Biosciences) were added to the wells. Cells were incubated for 5 hours at 37 °C and 5% CO_2_, after which NK cells were washed, stained with Ghost Dye™ Violet 510 (1:400, SKU 1-0870-T500, TONBO) and blocked with 20% rat serum, followed by staining with anti-mouse CD3e-FITC (5 µg/ml, clone 145-2C11, 35-0031-U500, TONBO) and NKp46-eFluor 450 (2 µg/ml, clone 29A1.4, 48-3351-82, ThermoFisher Scientific). Stained cells were fixed in 3% formaldehyde, permeabilized with Perm/Was buffer (554723, BD Biosciences) and stained intracellularly with anti-mouse IFN-γ-APC (5 µg/ml, clone XMG1.2, 20-7311-U100, TONBO). Cells were analyzed using an Attune NxT flow cytometer (Thermo Fisher Scientific) and FlowJo software version 10.8.1 (BD Biosciences).

### Inhibition analysis in ELISA

Sera were diluted to approximately 0.5 OD_405nm_, as determined in preceding serum ELISA titrations for coat HA-reactive antibodies, and incubated with various recombinant HA (5 µg/ml, H1, H2, H3, H4, H5, H6, H7, H9, H15, or H16) in binding buffer (PBS with 1% BSA and 0.2% tween 20) for 1 hour at room temperature. Serum-HA mixtures were transferred in triplicates to plates coated with recombinant H1, H7, H2, H3 or H5 (0.5 µg/ml) and incubated at room temperature for 1 h, before total IgG was detected as described above. OD_405nm_ from NaCl mice sera (diluted 1:50) was subtracted as background before calculating the relative inhibition (%) of OD_405nm_ signal of competed serum compared to serum that was pre-incubated with binding buffer alone (non-competed serum).

### Purification of H1-reactive antibodies from mouse serum

Recombinant H1 protein from PR8 (11684-V08H, Sino Biological) was coupled to CNBr-activated Sepharose 4 fast flow (1.5 mg protein:1 ml sepharose, 17098101, Cytiva). Antibodies were purified from 300 µl pooled mouse serum using the coupled sepharose in Pierce™ spin columns (69705, Thermo Fisher). After extensive washing of beads, antibodies were eluted with 1 column volume 0.1 M glycine HCl pH 2.7. The pH of the eluate was neutralized by adding 2 column volumes of 0.1 M glycine HCl pH 8.0. The glycine HCl solution was exchanged for PBS using three rounds of centrifugation with centrifugal filters with 10 kDa cut-off (UFC501, Merck), ending with a final volume of 100 µl.

### T cell ELISPOT

IFN-γ secreting T cells were analyzed using a Mouse IFN-γ ELISpot^PLUS^ kit (3321-4APT-2, Mabtech) according to the manufacturer’s protocol. Spleens or draining lymph nodes were harvested from vaccinated mice and dissociated in gentleMACS™ C Tubes. Splenocytes were treated with Tris-buffered ammonium chloride for 5 min on ice. Cells were filtered through a 70 nm Nylon strainer before counting, and 5 × 10^5^ viable splenocytes or 2.5 × 10^5^ draining lymph node cells per well in a complete medium were seeded in the pre-coated ELISPOT plates. Cells were re-stimulated for 20 hours at 37 °C at 5% CO_2_ with overlapping HA peptides from H1N1 viruses PR8 (PM-INFA-HAPR) or Cal07 (PM-INFA-HACal, 15-mers with 11 aa overlap, 0.1 µg/peptide/ml, JPT Peptide Technologies GmbH), or with 2 µg/ml H1 peptide (aa 532–540, IYSTVASSL, K^d^, GenScript). Spots were counted automatically using an ImmunoSpot® CORE cell counter (Cellular Technology Limited). For each sample, spot counts from wells stimulated with 2 µg/ml of an irrelevant peptide (aa 91-107, ALWFRNHFVFGGGTKVT, of the λ2 chain of the M315 mouse myeloma protein, GenScript) were subtracted as background.

### Viral challenge with influenza virus

BALB/c mice were anaesthetized as described under DNA vaccination. H1N1 PR8 virus, H1N1 Cal07 virus or A/turkey/Italy/3889/1999 (H7N1) virus stocks were diluted to either 2.5xLD_50_ or 5xLD_50_ in PBS as described per experiment, and administered in 10 µl to each nostril. Weight loss was monitored, and 20% weight loss was used as humane endpoint as approved by the Norwegian Animal Research Authority. Mice that reached this endpoint were euthanized and excluded from the weight curves from that point on.

### T cell depletion

BALB/c mice were treated with intraperitoneal injections with 100 µl PBS with 100 µg of both purified anti-CD4 (clone GK1.5, produced from hybridoma TIB207, ATCC) and anti-CD8 (clone 53-6.72, produced from hybridoma TIB105, ATCC), or with isotype matched controls (clone SFR8-B6 produced from HB-152, and clone Y13-238 produced from CRL-1741, both from ATCC), starting two days before challenge. Injections were repeated every second day until the end of the experiment. Mice were challenged with 5xLD_50_ H1N1 PR8 virus, as described above.

### Statistical analysis

Results were graphed and analyzed for significance using GraphPad Prism 8 software. Antibody titers and data from T cell ELISPOT and flow cytometry analysis were compared using Kruskal-Wallis with Dunn’s multiple comparisons test. Protein expression by transfected HEK239E cells and weight loss at day 4-6 after viral challenges were compared between groups using one-way ANOVA with Dunnet’s multiple comparisons test. Complete weight loss curves after infection were compared using two-way ANOVA. Survival curves were analyzed for significance using the Mantel–Cox test. In inhibition ELISA, correlations between % homology of competing HA with coat HA and % inhibition was analyzed with the Pearson correlation coefficient. *P* values < 0.05 were considered as statistically significant.

### Reporting summary

Further information on research design is available in the Nature Portfolio Reporting Summary linked to this article.

### Supplementary information

Supplementary Information

Reporting Summary

### Source data

Source data

## Data Availability

Data and material not included in the manuscript supplementary material will be made available to qualified academic researchers upon request to the corresponding author. The data used to generate the main results shown in the main figures and Supplementary Information are available as Source data. Source data are provided with this paper.
