# Supplementary material for: Applying valency-based immuno-selection to generate broadly cross-reactive antibodies against influenza hemagglutinins
Source: Nat Commun. 2024 Feb 12;15:850. doi: 10.1038/s41467-024-44889-w (PMC10861589; doi:10.1038/s41467-024-44889-w)
Supplement: Supplementary file 1 — Supplementary Information [file 41467_2024_44889_MOESM1_ESM.pdf]

Supplementary information for

Applying valency-based immuno-selection to generate broadly cross-reactive antibodies against influenza hemagglutinins

Daniëla Maria Hinke[1,2], Ane Marie Anderson[1,2], Kirankumar Katta[2], Marlene Fyrstenberg Laursen[2], Demo Yemane Tesfaye[2], Ina Charlotta Werninghaus[2], Davide Angeletti[3], Gunnveig Grødeland[1,2], Bjarne Bogen[1,2,4,*], and Ranveig Braathen[1,2,4,*]

[1]*K.G. Jebsen Centre for Influenza Vaccine Research, University of Oslo, Oslo, Norway*

[2]*Institute of Immunology (IMM), University of Oslo and Oslo University Hospital, Oslo, Norway*

[3]*Department of Microbiology and Immunology, Institute of Biomedicine, University of Gothenburg, Gothenburg, Sweden.*

[4]These authors jointly supervised this work

*Correspondence should be addressed to PhD Ranveig Braathen or to Prof. Bjarne Bogen, K.G. Jebsen Centre for Influenza Vaccine Research, Institute of Clinical Medicine, University of Oslo and Oslo University Hospital, 0027, Oslo, Norway. E-mail addresses: ranveig.braathen@medisin.uio.no (RB) or bjarne.bogen@medisin.uio.no (BB)

This PDF file includes:

Supplementary Table I

Supplementary Figure 1 to 16

Supplementary Table I. Overview category numbers of commercially obtained codon optimized HA DNA and recombinant HA and HA1 subunit proteins used in the manuscript

HA subtype	Virus strain	Cat# codon optimized cDNA*	Cat# rHA/HA1 protein*
H1	H1N1 (A/Puerto Rico/8/1934) (PR8)		HA: 11684-V08H (HA)<br>HA1: 11684-V08H1
	H1N1 (A/California/07/2009) (Cal07)		HA: 11085-V08H
H2	H2N2 (A/Canada/720/2005)	VG11688-UT	HA: 11688-V08H
H3	H3N2 (A/HongKong/1/1968)	VG40116-UT	HA: 40116-V08B
H4	H4N6 (A/Swine/Ontario/01911-1/1999)	VG11706-UT	HA: 11706-V08H
H5	H5N1 (A/Hong Kong/483/1997)		HA: 11689-V08H
H6	H6N1 (A/northern shoveler/California/HKWF115/2007)		HA: 11723-V08H
H7	H7N9 (A/Shanghai/1/2013)		HA: 40104-V08H<br>HA1: 40104-V08H1
H8	H8N4 (A/pintail duck/Alberta/114/1979)		HA: 11722-V08H
H9	H9N2 (A/Hong Kong/1073/1999)		HA: 11229-V08H
H10	H10N3 (A/duck/Hunan/S11205/2012)	VG40360-UT	HA1: 40360-V08H1**
H11	H11N2 (A/duck/Yangzhou/906/2002)		HA: 11705-V08H
H12	H12N5 (A/green-winged teal/ALB/199/1991)	VG11718-UT	HA: 11718-V08H
H13	H13N8 (A/black-headed gull/Netherlands/1/2000)		HA: 11721-V08B
H14	H14N5 (A/Mallard/Astrakhan(Gurjev)/263/1982)	VG40192-UT	HA: 40192-V08B
H15	H15N8 (A/duck/AUS/341/1983)	VG11720-UT	HA: 11720-V08H
H16	H16N3 (A/black-headed gull/Sweden/5/1999)	VG11711-UT	HA: 11711-V08H
H17	H17N10 (little yellow-shouldered bat/Guatemala/164/2009)	VG40323-UT	HA: 40323-V08H1
H18	H18N11 (A/flat-faced bat/Peru/033/2011)	VG40324-UT	HA: 40324-V08B

* Both codon optimized cDNA, and recombinant HA and HA1 proteins were ordered from Sino Biological
** For H10, we only found the HA1 subunit available at the time of our experiments

a

```
        18                                                                                107
H1_PR8  DTICIGYHANNSTDTVDTVLEKNVTVTHSVNLLEDSHNGKLCRLKGIAPLQLGKCNIAGWLLGNPECDPLLPVRSWSYIVETPNSENGIC
H1_C07  ..L..............................K.....K.R.V..H.........I.....ES.STAS.........S.D..T.
H2      .Q..........EK..I..R.....AKDI..KT.....K.N..P..E..D.S............R..S.PE....M.KE.PRD.L.
H5      .Q..........EQ..IM.......AQDI..RT.....D.N.VK..I.RD.SV........M..EFIN.PE......KASPA.DL.
H6      .K..........TQ..I..........E...NQKEERF.KILNK...D.RG.T.E..I....Q..L..GDQ.......R.TAQ....
H11     .E.....LS.....K...II.N.....S..E.V.TE.T.SF.SIN.KQ.IS..D.SF...I....M..D.IGKT.......K..PT....
H9      .K....HQST...E....LT.T..P...AKE..HTE...M..ATSLGH..I.DT.T.E.LVY...S..L..GG.E......RSSAV..T.
H8      .R.....QS.......N.LI.Q..P..QTME.V.TEKHPAY.NTDLG...E.RD.K.EAVIY...K..IH.KDQG......R.SAPE.M.
H13     .R..V..LST..SER...L..N..P..S..D.V.TN.T.TY.S.G..S.VH..D.SFE..IV...A.ASN.GI.E...LI.D.SAPH.L.
H17     .R.....Q..QNNQ..N.L..Q..P..GAQEI..TN......S.N.VP..D.QS.TL........N..S..EAEE....KINESAPDDL.
H16     .K.....LS..A......LT.NG.P..S..D.V.TN.T.TY.S.N..S.IH..D.SFE..IV...S.ATNINI.E...LI.D..AP.KL.
H18     .Q......S....Q..N.L..S..P..S.HSI..KE...L..K...K...D.ID.SLPA..M...K..E..TASE.A..K.D.EP.....
H12     .K.....QT....E..N.LI.Q..P..QVEE.VHGGIDPI..GTELGS..V.DD.SLE.LI....K..LY.NG.E......R.KEME.V.

        108                                                                               187
H1_PR8  YPGDFIDYEELREQLSSVSSFERFEIFPKESSWPNHN-TN-GVTAAC-SH-EGK-SSFYRNLLWLTE-KE-GSYP---KLKNSYVNKKGK
H1_C07  ...........................T.....D-S.K......-P.-A.A-K...K..I..VK-.G-N..---..SK..I.D...
H2      ...S.N.....KHL....KH..KVK.L..DR-.TQ.T-.T-.GSR..-AV-S.N-P..F..MV...K-.G-SN..---VAQG..N.TS.E
H5      ...N.N.....KHL..RI.H..KIQ.I..-...S..DASS-..SS..-PY-L..-...F..VV..IK-.N-ST..---TI.R..N.TNQE
H6      ...ALNEV...KALIG.GERV....M...-.T.TGVD-.SS...K..-PY-NSG-.........IIKT.S-AA..---VI.GT.N.TGSQ
H11     ...ILENE....LKF.G.LE.NK..A.TSNGWGAVNS-GA-......-KF-GSS-N..F..MI..IH-QS-.T..---VI.RTFN.T..R
H9      ...NVENL....TLF..A..YQ.IQ...-DTT.-.VT-YT-.TSR..-.------G....SMR...Q-.S-.F..---VQDAQ.T.NR..
H8      ...SIENL....FVF..AA.YK.IRL.-DY.R.-.VT-RS-.TSK..NAS-T.G-Q....SIN...K-.KPDT.D---FNEGT...NEDG
H13     ...ELDNNG...HLF.GIR..S.T.LIAP-T..GAV.--D-..SS..-QD-K.A-.......V.FV.-RG-NK..---VIRGT.N.TT.R
H17     F..N.ENLQD.LLEM.G.QN.TKVKL.NPQ.-MTGVT-..-N.DQT.-PF-...-P......N.IQG-NS-.-L.FNIEI..PTS.P---
H16     F..ELDNNG...HLF.G.N..S.T.LISP-NK.GDI--LD-....S.-RD-N.A-.......V.IVKN.N-.K..---VI.GD.N.TT.R
H18     F....DSL.D.ILLV.NTDH.RKEK.IDM-TRFSDVT-..-N.DS..-PY-DTNGA......N.VQQ-NK-.K-----Q.IFH.Q.SENN
H12     ...SIENQ....SLF..IKKY..VKM.-DFTK.-.VT-YT-.TSK..-NNTSNQ-G....SMR...L-.S-.QF.---VQTDE.K.TRDS

        188                                                                               276
H1_PR8  EVLVLWGIHHPPNSKEQQNLYQNENAYVSVVTSNYNRRFTPEIAERPKVRD-QAGRMNYYWTLLKPGDTIIFEANGNLIAPMYAFALSRG
H1_C07  ...........ST.AD..S....AD...F.GS.R.SKK.K....I......-RE.........VE...K.T...T...VV.R....ME.N
H2      QM.II..V...NDET..RT....VGT....G..TL.K.S.....T....NG-.G...EFS....DMW...N..ST......E.G.KI.KR
H5      DL.........NDAA..TK....PTT.I..G..TL.Q.LV....T....NG-.S..IEFF..I...N.A.N..S...F...E..YKIVKK
H6      PI.YF..V....DTN..NT..GSGDR..RMG.ESM.FAKS....A..A.NG-.R..ID...SV....E.LNV.S.......W..YKFVST
H11     D...V......ATL..H.D..KKDSS..A.GSET.........ST....NG-.....TF...IV...ES.T..S..AFL..R....EIVSV
H9      SI.FV.......TYT..T...IRNDTTT..T.EDL..T.K.V.GP..L.NG-LQ..ID...SV....Q.LRVRS.......W.GHV..G.
H8      DIIF........DT...TT..K.A.TLT..T.NTI..N.Q.N.GP..L..G-.Q...D...GI..R.E.LKIRT.......EFGYL.-K.
H13     D...I......VSTD.ARK..V.D.P.TL.S..SWS.KYNL..GI..GYNG-.KSW.KI..Y.MH..ES.S..S..G.L..K.GYIIEE-
H17     -L.L.....NTKDAAQ.R...G.DYS.TIFNFGEKSEE.R...GQ.DE.KA-HQD.ID...GS.PAQS.LRI.ST......E.G.YYK.K
H16     D..........DTETTAI...ASK.P.TL.S.KEWSK.YEL..GT.--IG.G.RSW.KL..H.MR..ER.M..S..G....R.GYIIEK-
H18     PL.II..V.QTS.AA..NTY.GSQTGSTTITIGEETNTYPLV.S.SSILNG-HSD.I..F.GVVN.NQNFSIVST..F.W.E.GYFFQKT
H12     DIVFT.A.....T.D..VK..K.PDTLS..T.DEI..S.K.N.GP..L..G-.Q...D...AV....Q.VKIQT.......E.GHLITGK

        277                                                                 HA1\/HA2      358
H1_PR8  FG-SG-IITSNASM--HECNTKCQTPLGAINSSLPYQNIHPVTIGECPKYVRSAKLRMVTGLRNIPSIQ----SRGLFGAIAGFIEGGWT
H1_C07  A.-..-..I.DTPV--.D...T....K....T...F.....I...K.....K.T...LA.........----...............
H2      GS-..-.MKTEGTL--EN.E...........TT..FH.V..L.........K.E..VLA.....V.Q.E----...............Q
H5      GD-.T-.MK.ELEY--GN.........M......M.FH....L.........K.NR.VLA.....A.QRERRRKK..............Q
H6      NN-K.A.FK..LPI--EN.DAT...IA.VLRTNKTF..VS.LW.........K.ES..LA.....V.Q.E----T...............
H11     GN-GK-LFR.ELNI--ES.S.....EV.G..TNKSFHSV.RN...D.....NVKS.KLA.....V.A.A----...............P
H9      SH-GR-.LKTDLKG--GN.VVQ...EK.GL..T..FH..SKYAF.T......VNS.KLAV....V.ARS----...............P
H8      ES-H.-R.IQ.EDIPIGN.......YA......K.F..ASRHYM.......KK.S..LAV....T..VE----P...............S
H13     Y.-K.R.FQ.RIRI--AK.......SV.G..TNKTF...ERNAL.D....IK.GQ.KLA.....V.A.S----K...............P
H17     E.-K.GLMK.KLPI--SD.S.........L..T..F..V.QQ...N.....KATS.MLA.....N.QME----G...............Q
H16     Y.-T.R.FQ.GVR.--AK.......S..G..TNKTF...ERNAL.D....IK.GQ.KLA.....V..VG----E...............P
H18     TNI..-..K.SEKI--SD.D.I...KI.....T..F....QNA..D.....KAQE.VLA.....N.IKE----T...............Q
H12     SH-GR-.LKN.LPI--GQ.V.E..LNE.VM.T.K.F..TSKHY..K....IP.GS.KLAI....V.QV.----D...............P
```

```
       359                                                                                    448
H1_PR8 GMIDGWYGYHHQNEQGSGYAADQKSTQNAINGITNKVNTVIEKMNIQFTAVGKEFNKLEKRMENLNKKVDDGFLDIWTYNAELLVLLENE
H1_C07 ..V...................L.......DE......S......T..........H....I............................
H2     ..V........S.D........KE...K.FD.......S......T..E......SN..R.L......ME.....V..........M...
H5     ..V........S...........E...K..D.V.....SI.N...T..E...R...N..R.I......ME.....V..........M...
H6     ...........E.S........KE...K..D.......SI.D...T..E..DH..SN..R.ID....RME.....V..............
H11    .L.N....FQ.R..E.T.I...KE...K..DQ..S...NIVDR..TN.ES.QH..SEI.E.INR.S.H...SVV...S...Q........
H9     .LVA....FQ.S.D..V.M...RD...K..DK..S...NIVD...K.YEIIDH..SEV.T.LNMI.N.I..QIQ.V.A...........Q
H8     ........F..S.SE.T.M........E..DK......NIVD...RE.EV.NH..SEV...INMI.D.I..QIE.L.A...........Q
H13    .L.N....FQ......V.M...KE...K..DQ..T.I.NI.....GNYDSIRG..SQV.Q.INM.ADRI..AVT.V.S...K.......D
H17    ...........E.QE.......KEA..K.VDA......SI.D...S..ESNI....R..L.IQH.SDR...AL....S..T.........
H16    .L.N....FQ......T.I...KA...K..DE..T.I.NI.....GNYDSIRG...QV...INM.ADR...AVT...S...K.......G
H18    .L...........SE.......KEA..K.VDA..T...NI.D...T..ESTA.....I.M.IKH.SDR.......V.S............
H12    .LVA....FQ...AE.T.I...RD...K..DNMQ..L.N..D...K..EV.NH..SEV.S.INTI.S.I..QIT...A...........Q

       449                                                                                    535
H1_PR8 RTLDFHDSNVKNLYEKVKSQLKNNAKEIGNGCFEFYHKCDNECMESVRNGTYDYPKYSEESKLNREKVDGVKLES-M-G-IYQILAIYST
H1_C07 ....Y............R.......................T.....K............A.....EI.......-T-R-..........
H2     ..............D..RM..RD.V..L............D...N..K.........E.......NEIK....S.-.-.-V.......A.
H5     ..............D..RL..RD....L...................K.......Q....AR....EIS......-.-.-T....SL...
H6     ....L..A.............RD..NDL.......W.......I...K.........QD......QEIES...DN-L-.-V.........
H11    K...L.....R..H.R.RRM..D...DE.....T.........I.K.......HKEFEK...I..QEIE....D.-S-.NV.K..S...C
H9     K...E..A..N...N...RA.GS..M.D.K....L.....DQ...TI.....NRR..R...R.E.Q.IE......-E-.-T.K..T....
H8     K...E........FDE..RR.SA..IDA.....DIL.........TIK....NHKE.E..A..E.S.IN.....E-N-T-T.K..S....
H13    K...M..A..R..HDQ.RRV..T..IDE......LL...NDS...TI.....NHTE.E.....K.QEIE.I..K.DD-S-V.KA.S...C
H17    .......A.....F....A...D..IDE.....LLL...N.S..DDIK....K.MD.R...HIEKQ.I.....TD-Y-S-R.Y.MTL...
H16    ....L..A..R..HDQ..RI..S..IDE.D...NLL...NDS...TI.....NHED.R...Q.K.QEIE.I..K.-EDN-V.KV.S...C
H18    .......A..N...Q...V...D..IDM.....KIL...N.T..DDIK....N.YE.RK..H.EKQ.I.....SE-N-S-S.K.MI....
H12    K...E..A..R..HDR.RRV.RE..IDT.D....IL....DN..DTI.....NHKE.E....IE.Q.IN.....E-N-S-T.K..S...S
                                                                                             ====
         540
H1_PR8 VASSL
H1_C07 .....
H2     ..G..
H5     ....
H6     .S...
H11    I....
H9     .....
H8     ..A..
H13    I...I
H17    I...V
H16    I...I
H18    ....V
H12    .....
       ===== CD8 T cell epitope
```

b

```
     19                                                                                     108
H7   DKICLGHHAVSNGTKVNTLTERGVEVVNATETVERTNVPRICSKGKRTVDLGQCGLLGTITGPPQCDQFLEFSADLIIERREGSDVCYPG
H15  ..........A..........K............I.GINKV.T...KA....S..I....I......SH.K.K........NS..I....
H10  ..........A...T.K...NEQE..T.......SIGLN.L.M..RNHK...N.HPI.MLI.T.A..LH.TGTW.TL...ENAIAY....
H14    ........E...S.K...DNH....S.K.L..TNHTDEL.PSPLKL..GQD.H.INGAL.S.G..RLQDTTW.VF...PTAV.T...F
H3     L.......P...L.K.I.DDQI..T....L.QSSSTGK..NNPH.IL.GID.T.IDALL.D.H..V.QNETW..FV..SKAFSN...Y
H4     ............M.K...DDQI...T.Q.L..SQHL.EL.PSPL.L..GQT.DIVNGAL.S.G..HLNGAEW.VF...PTAV.T...F

     109                                                                                    195
H7   KFVNEEALRQILRESGGIDKEAMGFTY-SGIRTNGTTSTCRR-SG-SSFYAEMKWLLSNTDNAAFPQMTKSYKNTRKDPALIIWGIHHSG
H15  ..T........I.........P...R.-...K.D.A..A.K.-TV-....S.......SKA.QV...LNQT.R.N..E....V..V...S
H10  AT........KIM.....N.IST....G.S.NSA...RA.M.-N.GN.....L...V.KSKGQN...T.NT.R..DTAEH..M.....PS
H14  DVPDYQS..S..AS..SLEFI.EQ..W-N.VKVD.SS.A.L.-G.RN..FSRLN..TKA.-.GNYGPINVTKE..GSYVR.YL..V..PS
H3   DVPDYAS..SLVAS..TLEFITE...W-T.VTQ..GSNA.K.GP.-.G.FSRLN..TKS--GSTY.VLNVTMP.NDNFDK.Y...V..PS
H4   DVPDYQS..S..ANN.KFEFI.EE.QW-NTVKQ..KSGA.K.-ANVND.FNRLN..-TKS.GN.Y.LQNLTKV.NGDYAR.Y...V..PS

     196                                                                                    272
H7   STTEQTKLYGSGNKLITVGSSNYQQSFVPSPGERPQVNGQSGRIDFHWLMLNPNDTVTFSFNGAFIAPDRASFL--R----------GKS
H15  .LD..N....A..........K.....S....D..K....A.......ML.D.G.....T...........T..--.SNAPSGVEYN...
H10  ..Q.KND...TQSLS.S....T..NN...VV.A...............TLVQ.G.NI...H..GL...S.V.K.--I----------.RG
H14  .DN...D..KVATGRV..STRSD.I.I..NI.S..R.RN.....SIY.TLV..G.SII.NSI.NL...-.GHYK--I----------S..
H3   TNQ...S..VQASGRV..STRRS..TII.NI.S..W.R.L.S..SIY.TIVK.G.VLVINS..NL...RGYFKM--.T---------...
H4   TD....N..KNNPGRV..STQTS.T.V..NI.S..W.R.L.S..S.Y.TIVE.G.LIV.NTI.NL...RGHYK.NSQ----------K..

                                                                     (ΔMBS)
     273                                                              HA1\/HA2              356
H7   MG---IQSGVQVDANCEGDCYHSGGTIISNLPFQNINSRAVGKCPRYVKQESLLLATGMKNVPEIPKGSGLFGAIAGFIENGWEGLIDGW
H15  L.---...DA.I.ES...E.FY.....N.P......D.W...R.......S..P..L.......KIHTR.....................
H10  L.---...DAPI.N...SK.FWR..S.NTR.....LSPKT..Q..K..NKK..M.....R....LMQ.R................MV...
H14  TKSTVLK.DKRI-GS.TSP.LTDK.S.Q.DK....VSRI.I.N..K....G..M.....R.I.G-KQAK..............Q......
H3   SI---MR.DAPI.T-.ISE.ITPN.S.PNDK....V.KITY.A..K....NT.K.....R....-KQTR................M....
H4   TI---LNTA.PI-GS.VSK.HTDK.S.STTK.....SRISI.D..K....G..K.....R.I..-KATR..............Q......

     357                                                                                    446
H7   YGFRHQNAQGEGTAADYKSTQSAIDQVTGKLNRLIEKTNQQFELIDNEFTEVEKQIGNVINWTRDSMTEVWSYNAELLVAMENQHTIDLT
H15  ..........Q..........A....I............T.............Q............L..I...................A
H10  ..........T.Q........AV...I............TE..S.ES..S.I.H.........K..I.DI.T.Q..............MA
H14  ........E.T.....L....A....IN...........EKYHQ.EK..EQ..GR.QDLEKYVE.TKIDL..........L.......V.
H3   .......SE.T.Q...L....A....IN.....V.....EK.HQ.EK..S...GR.QDLEKYVE.TKIDL..........L.........
H4   ........E.T.....L....A....IN.........P.EKYHQ.EK..EQ..GR.QDLEKYVE.TKIDL..........L.......V.

     447                                                                            536
H7   DSEMNKLYERVKRLLRENAEEDGTGCFEIFHKCDDDCMASIRNNTYDHSKYREEAMQNRIQIDPVKLSSGYKDVILWFSFGA
H15  ...........R.Q.................R...Q..E.......N.TE..Q..L....M.N.....G.............
H10  ....LN.....RKQ..Q......K.....Y.A...S..E..........Q.....LL..LN.N..T.......I........
H14  .......F...R.Q......DQ.N.......Q..NN.IE....G....NI..D..IN...K.N..T.TM....I...I..
H3   .......F.KTR.Q......DM.N...K.Y....NA.IE....G....DV..D..LN..F..KG.E.K.....W...I..A
H4   .......F...RHQ......DK.N.......Q..NS.IE....G....DI..D..IN..F..QG...IQ....I......
```

c

```
        18                                                                                     107
H1_PR8  DTICIGYHANNSTDTVDTVLEKNVTVTHSVNLLEDSHNGKLCRLKGIAPLQLGKCNIAGWLLGNPECDPLLPVRSWSYIVETPNSENGIC
H7      .K..L.H..VSNGTK.N.LT.RG.E.VNATETV.RTNVPRI.S-..KRTVD..Q.GLL.TIT.P.Q..QF.EF-.ADL.I.RREGSD-V.
        19                                                                                     105

        108                                                                                    195
H1_PR8  YPGDFIDYEELREQLSSVSSFERFEIFPKESSWPNHNTNGVTAACSHEGKSSFYRNLLWLTE--KEGSYPKLKNSYVNKKGKEVLVLWGI
H7      ...K.VNE.A..QI.RESGGIDKEAM---GFTYSGIR...T.ST.RRS.-....AEMK..LSNTDNAAF.QMTK..K.TRKDPA.II...
        106                                                                                    191

        196                                                                                    285
H1_PR8  HHPPNSKEQQNLYQNENAYVSVVTSNYNRRFTPEIAERPKVRDQAGRMNYYWTLLKPGDTIIFEANGNLIAPMYAFALSRGFGSGIITSN
H7      ..SGSTT..TK..GSG.KLIT.GS...QQS.V.SPG...Q.NG.S..IDFH.LM.N.N..VT.SF..AF...DR.SF.-..KSM..QSGV
        192                                                                                    280

        286                                                    HA1\/HA2                        374
H1_PR8  ASMHECNTKCQTPLGAINSSLPYQNIHPVTIGECPKYVRSAKLRMVTGLRNIPSI-QSRGLFGAIAGFIEGGWTGMIDGWYGYHHQNEQG
H7      QVDAN.EGD.YHSG.T.I.N..F...NSRAV.K..R..KQES.LLA..MK.V.E.PKGS..........N..E.L......FR...A..
        281                                                   (ΔMBS)                            370

        375                                                                                    464
H1_PR8  SGYAADQKSTQNAINGITNKVNTVIEKMNIQFTAVGKEFNKLEKRMENLNKKVDDGFLDIWTYNAELLVLLENERTLDFHDSNVKNLYEK
H7      E.T...Y....S..DQV.G.L.RL...T.Q..ELIDN..TEV..QIG.VINWTR.SMTEV.S.......AM..QH.I.LT..EMNK...R
        371                                                                                    460

        465                                                     523
H1_PR8  VKSQLKNNAKEIGNGCFEFYHKCDNECMESVRNGTYDYPKYSEESKLNREKVDGVKLES
H7      ..RL.RE..E.D.T....IF....DD..A.I..N...HS..R..AMQ..IQI.P...S.
        461                                                     518
```

Supplementary Figure 1. Alignment of amino acid sequences of HA variants used in the HA mix vaccines. a-b Sequences of selected IAV group 1 (**a**) and group 2 (**b**) variants were aligned using multiple sequence alignment with blastp suite-2sequences (https://blast.ncbi.nlm.nih.gov/Blast.cgi?PROGRAM=blastp&PAGE_TYPE=BlastSearch&LINK_LOC=blasthome). Sequences are vertically arranged in the order of % shared amino acid identity with H1 of PR8 A/Puerto Rico/8/1934 (**a**, group 1) or H7 of A/chicken/Italy/13474/1999 (**b**, group 2). Amino acid numbering follows H1 PR8 (**a**) or H7 (**b**) numbering, respectively. The conserved fusion peptide in the HA stem is highlighted in gray. A conserved group 1 HA $CD8^{+}$ (K^{d}-restricted) T cell epitope in BALB/c is indicated underneath the sequences in **a**. **c** Comparison between group 1 (H1 of PR8) and group 2 (H7 of A/chicken/Italy/13474/1999) HA virus strains. The sequences were derived from the virus strains described in Table I. ΔMBS

indicates where the multibasic cleavage site (RVRR) was removed for H7. Amino acid sequences for each of the included HA proteins are provided as a Source Data file.

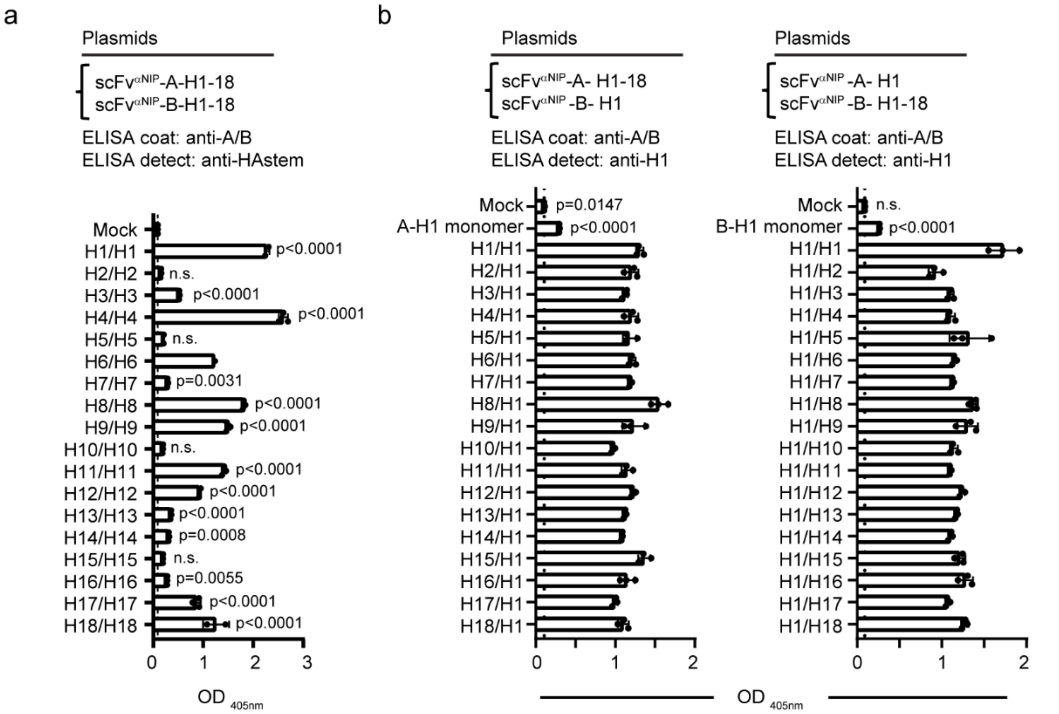

Supplementary Figure 2. Expression of HA subtypes on heterodimers with the non-targeting control, scFv$^{\alpha NIP}$. a HEK293E cells were transiently co-transfected with A and B plasmid pairs that encoded scFv$^{\alpha NIP}$ heterodimers bivalent for HA_x/HA_x for each of the 18 HA antigens (H1-18), indicated to the left of the graph (e.g. H1/H1). n=3, n=2 (H1/H1, H5/H5, H12/H12, and H18/H18), or n=1 (H6/H6) technical replicates. **b** Left: HEK293E cells were co-transfected with scFv$^{\alpha NIP}$-A plasmids expressing one HA out of the 18 HA subtypes (H1-18) and scFv$^{\alpha NIP}$-B-H1, resulting in monovalent HA_x/H1 heterodimers indicated to the left of the graph. Right: Vice versa, expressing H1 on the A arm and one of the 18 HA subtypes on the B arm (e.g. H1/HA_x). Supernatants from transiently co-transfected HEK cells were analyzed using sandwich ELISA with a combination of mAb towards the A/B motif and a broadly-reactive mAb towards the HA stem or anti-H1 mAb as indicated. Shown are mean of technical triplicates ± SD. All heterodimeric proteins in **b** were expressed in significantly higher amounts then the H1 monomer with $p<0.0001$. n.s. = not significant, one-way ANOVA with Dunnett's multiple comparisons,

comparing to expression of mock (**a**) or H1 monomer (**b**). Each panel presents data derived from n=1 independent experiment. Source data are provided as a Source Data file.

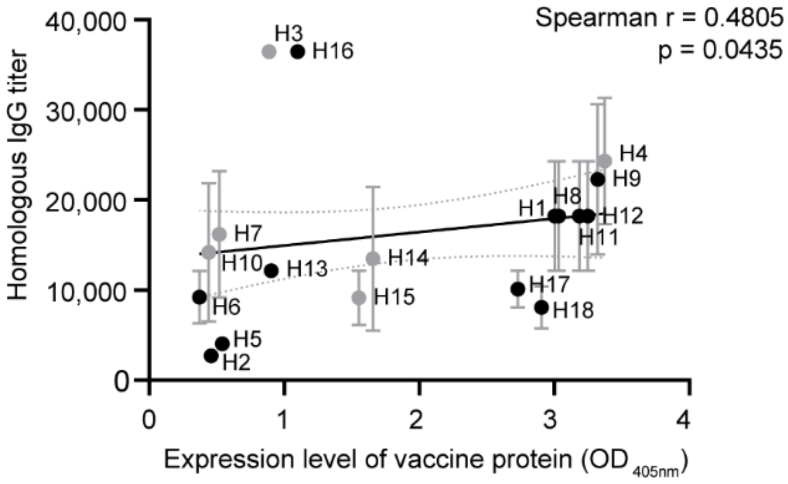

Supplementary Figure 3. Correlation between expression levels of HA_x/HA_x bivalent proteins from HEK293E cells and antibody levels induced after i.d. vaccination. HEK293E cells were transiently transfected with pairs of A and B plasmids that together encode heterodimers with two identical MHCII-specific targeting units and two identical HA antigenic units (HA_x/HA_x), for each of H1-18. Supernatants were analyzed in sandwich ELISA employing anti-A/B and anti-HA stem mAb (expression shown in Fig. 1d). Female BALB/cAnNRj mice were immunized with MHCII-targeted A/B plasmid pairs expressing bivalent HA subtypes for each of H1-18 (50 µg/plasmid, n=4/group) i.d./EP. Homologous HA subtype-specific serum IgG titers elicited by a given HA_x/HA_x six weeks after vaccination (vaccine reactivity shown in Fig. 2a) were correlated to the expression of corresponding MHCII-targeted bivalent HA_x/HA_x vaccines from transfected HEK293E cells. Shown are mean OD_{405nm} detected for each HA subtype in supernatant from transfected HEK293E cells in correlation to mean of HA subtype-specific serum IgG in serum from vaccinated mice. Black dots indicate group 1 HAs, gray dots indicate group 2 HAs. IgG serum titers are shown as mean ± SEM. Correlations were calculated using Spearman r correlation coefficients. Spearman r and p value are shown. Source data are provided as a Source Data file.

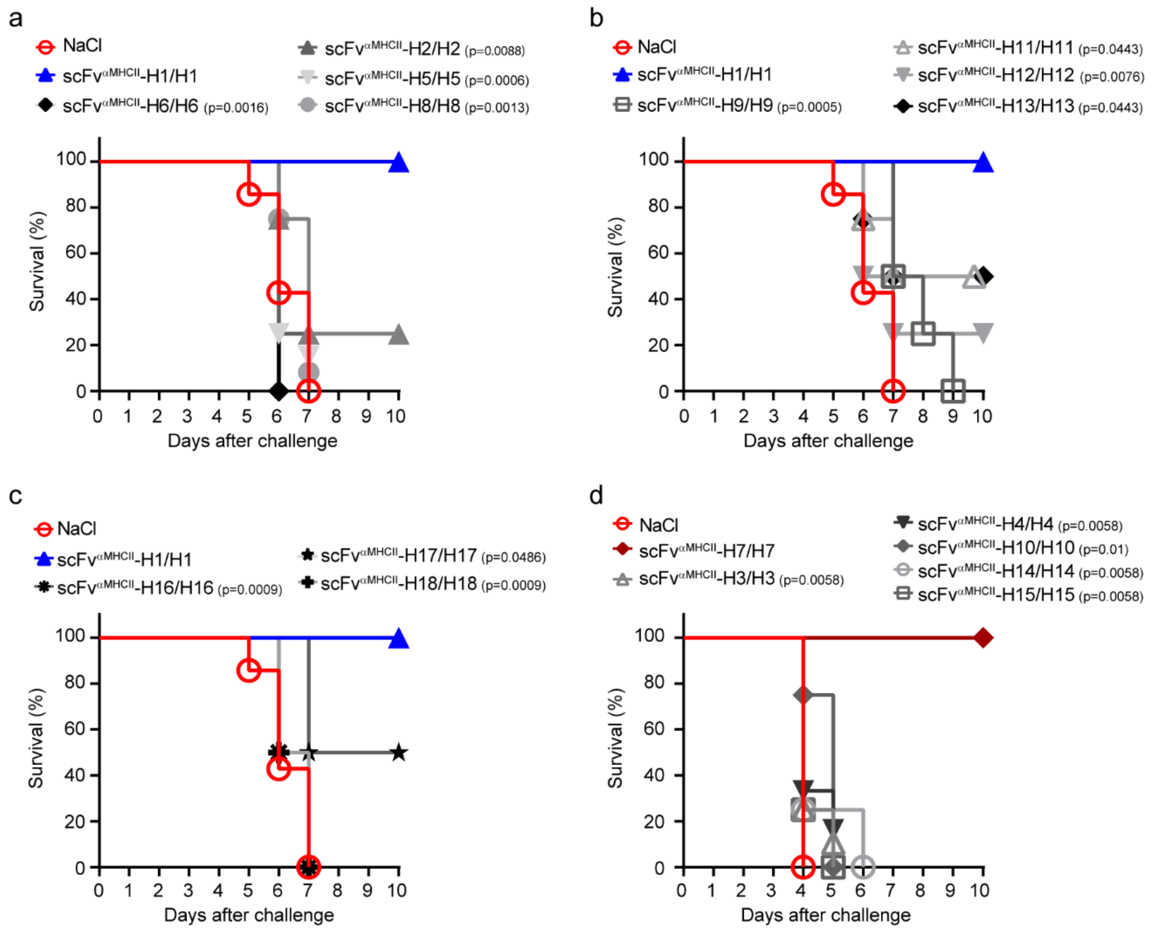

Supplementary Figure 4. Immunization with homologous H1/H1 or H7/H7 vaccines can protect mice from H1 or H7 challenge, respectively. a-d Female BALB/cAnNRj mice were immunized with the indicated vaccines encoding MHCII-targeted bivalent HA_x/HA_x for H1-H18 (50 µg/plasmid, n=4/group) i.d./EP. (**a-c**) Mice that had been immunized with indicated bivalent group 1 HAs were challenged with $5xLD_{50}$ PR8 (A/Puerto Rico/8/1934, H1N1) ten weeks after vaccination. The same NaCl control group (n=7 mice) and bivalent MHCII-targeted H1/H1 group (n=7 mice) were used in **a-c**. (**d**) Mice that had been immunized with indicated group 2 HAs were challenged with $5xLD_{50}$ A/turkey/Italy/3889/1999 (H7N1). Shown are survival curves. The corresponding weight curves are shown in Fig. 2 b-e. Within the legends, p values are indicated for survival of each immunization group compared to survival of H1/H1 (**a-c**) or H7/H7 (**d**), two-tailed Mantel Cox. Source data are provided as a Source Data file.

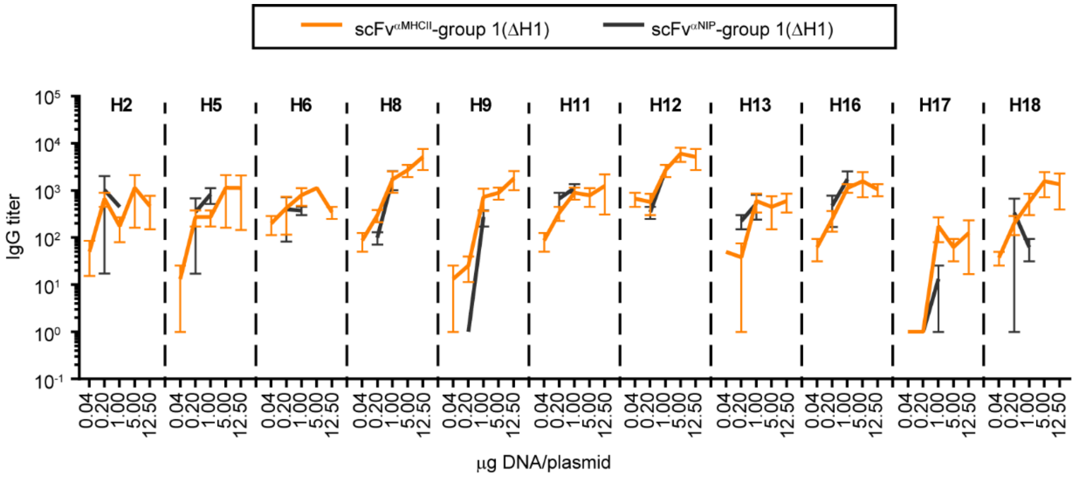

Supplementary Figure 5. Titration of plasmid mix vaccines for induction of HA-reactive antibodies. Female BALB/cAnNRj mice were vaccinated three times (weeks 0, 5 and 10, n=4/group) i.d./EP with the indicated µg DNA per plasmid. Mix vaccines contained scFv$^{\alpha MHCII}$-targeted or non-targeted (scFv$^{\alpha NIP}$) A/B plasmid pairs with HA subtypes from group 1 (except H1), with 6 HAs (H6, H8, H9, H11, H12 and H13) expressed on the A plasmid, and 5 HAs (H2, H5, H6, H18 and H17) expressed on the B plasmid. IgG antibodies against the HAs included in the mix were measured at week 6. Shown are mean ± SEM. n=1 independent experiment. Source data are provided as a Source Data file**.**

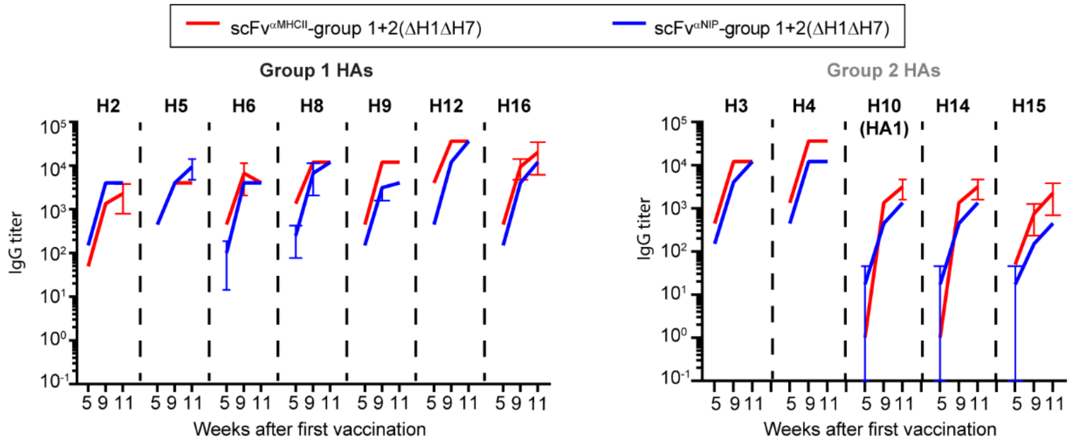

Supplementary Figure 6. **Intramuscular (i.m.) immunization with 16 HA subtypes in a plasmid mix vaccine induces antibodies against HAs included in the mix.** Female BALB/cAnNRj mice were vaccinated three times (week 0, 5 and 9) i.m./EP with a mixture of plasmids that encoded 16 HAs of both group 1 and group 2 (excluding H1 and H7, 1µg DNA/plasmid, n=6/group), n=1 independent experiment. The mix vaccine was either scFv$^{\alpha MHCII}$-targeted or non-targeted (scFv$^{\alpha NIP}$). Serum per vaccine group was pooled and IgG against group 1 and group 2 HAs was analyzed in technical triplicates. Shown are mean ± SD. Source data are provided as a Source Data file.

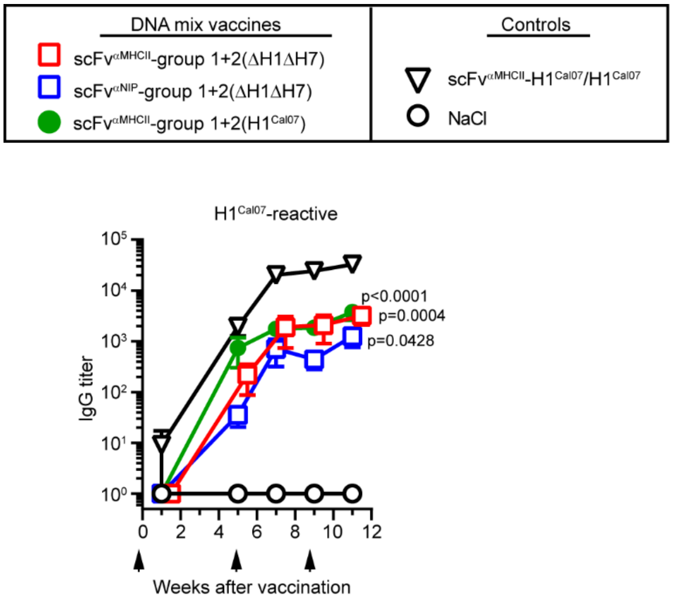

Supplementary Figure 7. Immunization with HA mix vaccines induces antibodies against Cal07 H1 protein. Female BALB/cAnNRj mice were immunized three times (indicated with arrows) i.m./EP with the indicated mix vaccines (1 µg DNA/plasmid, n=9 [scFv$^{\alpha MHCII}$-group1+2] or n=10/group [scFv$^{\alpha MHCII}$-group1+2(ΔH1ΔH7) and scFv$^{\alpha NIP}$-group1+2(ΔH1ΔH7)]) or with scFv$^{\alpha MHCII}$-targeted H1/H1 from Cal07 (5 µg DNA/plasmid, n=6) or NaCl as controls (n=8), n=1 independent experiment. The mix vaccine with 18 HA subtypes included H1 from Cal07 instead of PR8. Serum samples were harvested and analyzed for H1^{Cal07}-reactive IgG titers. Shown are mean titers ± SEM. Significance is calculated using Kruskal-Wallis with Dunn's multiple comparisons test comparing titers at the final time point to NaCl-induced titers. Source data are provided as a Source Data file.

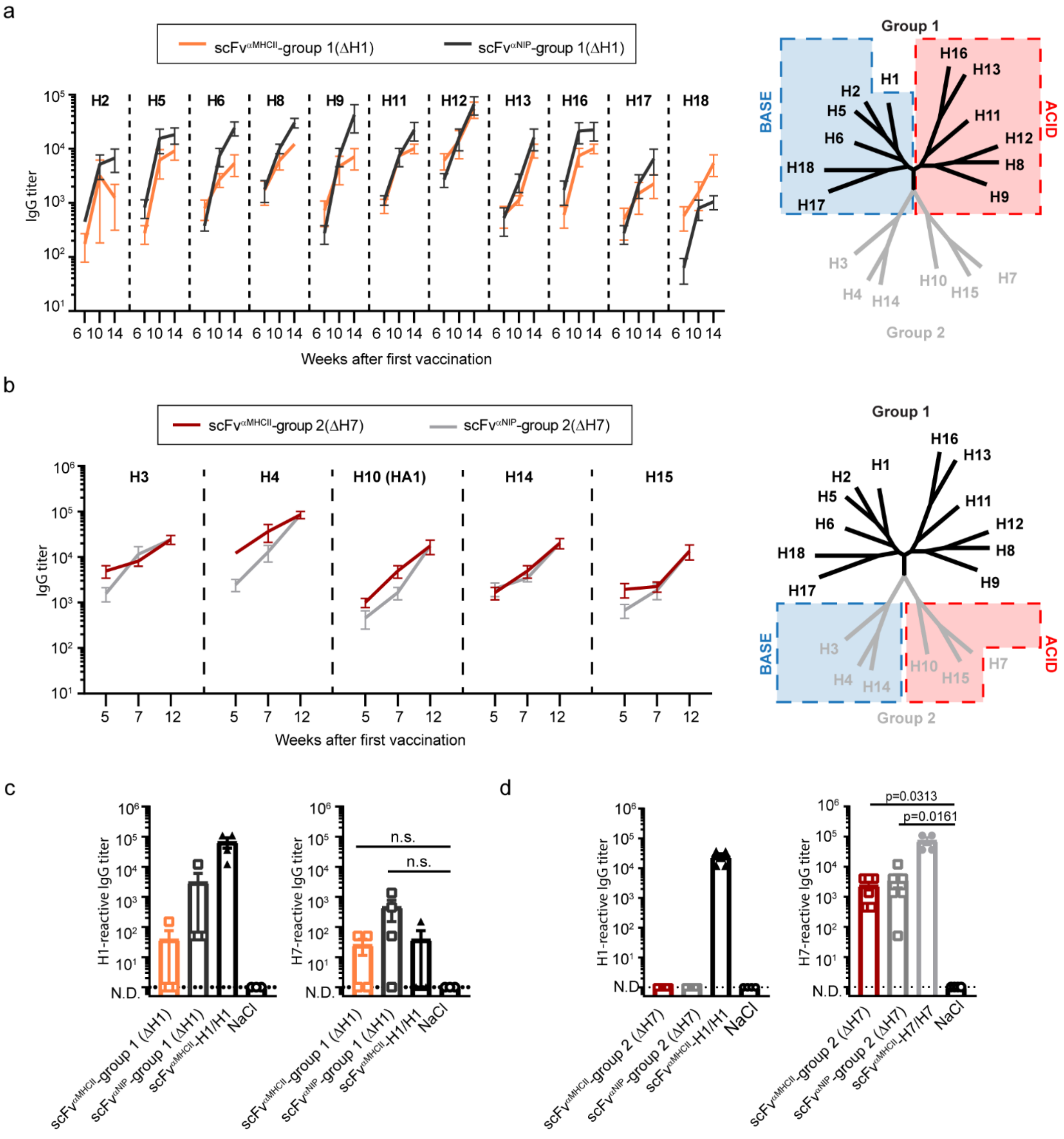

Supplementary Figure 8. Immunization with distinct group 1 and group 2 HA plasmid mixes induce antibodies against HAs included in the mix as well as against non-included HAs of the same group. a Female BALB/cAnNRj mice were vaccinated three times (weeks 0, 6 and 11) i.d./EP (1 µg DNA/plasmid, n=4/group) with plasmid mixes that contained HA subtypes from group 1 (except H1) or with scFv$^{\alpha MHCII}$-targeted bivalent H1/H1 (5 µg DNA/plasmid, n=4)

or NaCl (n=4) as controls. The included HA subtypes were expressed with either A or B motifs as indicated on the right. The mix vaccines were either MHCII-targeted (scFv$^{\alpha MHCII}$) or non-targeted (scFv$^{\alpha NIP}$). IgG antibodies were measured at the indicated time points against the HAs included in the mix. Shown are mean ± SEM. **b** As in **a**, but with plasmid mixes of group 2 HAs except H7 (n=6/group), or with scFv$^{\alpha MHCII}$-targeted bivalent H7/H7 (n=4) or NaCl (n=4) as controls. **c-d** Sera from Exp. **a** at week 16 (**c**) and Exp. **b** at week 12 (**d**) were tested for IgG antibodies against PR8 H1 and H7 (not included in the mixes). Mean titers ± SEM are shown. Significance is calculated using Kruskal-Wallis with Dunn's multiple comparisons test. N.D. = non-detectable, n.s. = not significant. Each panel presents data derived from n=1 independent experiment. Source data are provided as a Source Data file.

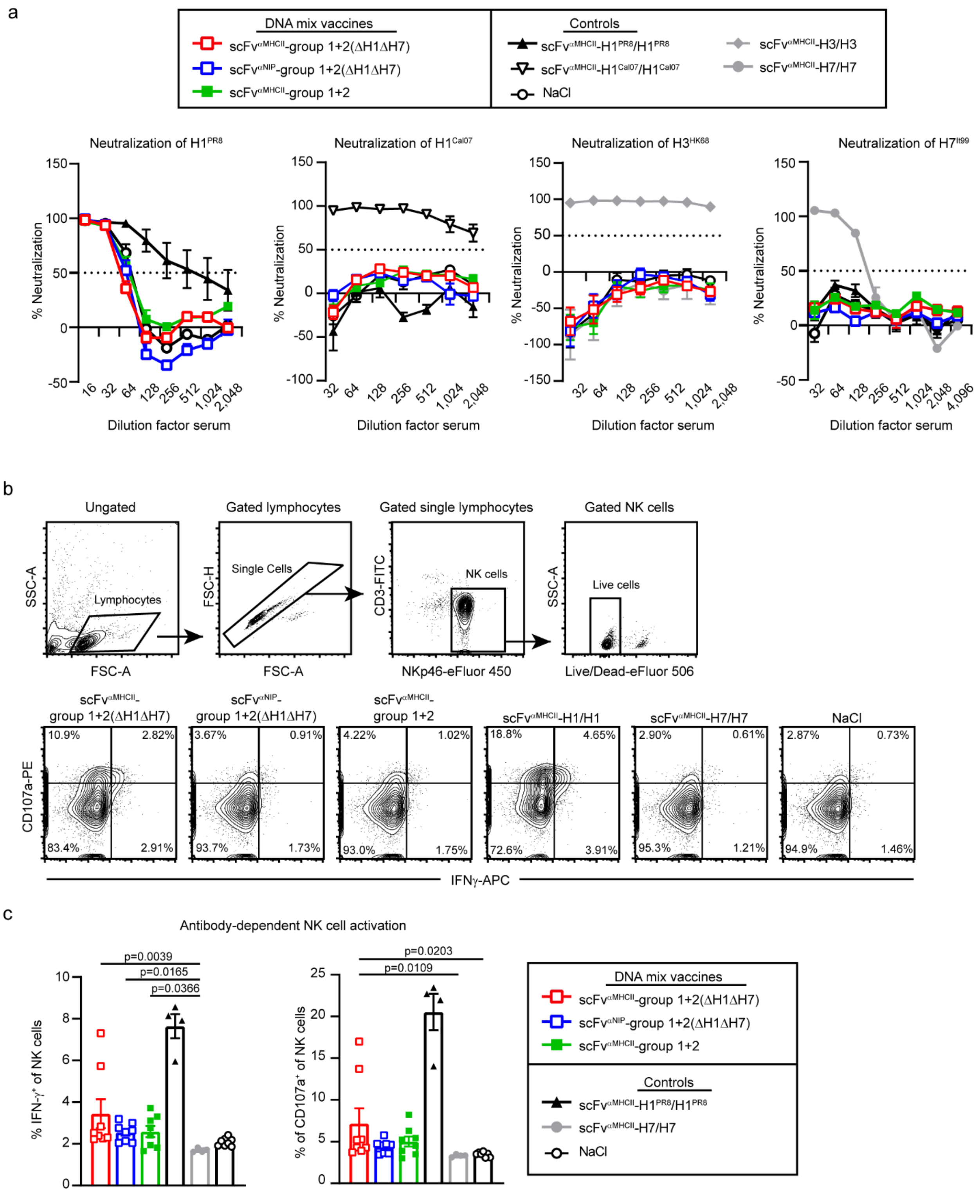

Supplementary Figure 9. Neutralization and antibody-dependent NK cell activation by sera

from mix-vaccinated mice. a-c Female BALB/cAnNRj mice were immunized three times (week 0, 5 and 9) i.m./EP with the indicated plasmid mix (1 µg DNA/plasmid, n=8/group), bivalent control vaccines (MHCII-targeted H1/H1 [n=4], H3/H3 [n=6] or H7/H7 [n=4], 5 µg DNA/plasmid), or NaCl control (n=8). Serum was harvested two weeks after final vaccination (week 11). (**a**) In vitro neutralization of either PR8, Cal07, H3 [H3N2(A/HongKong/1/1968)] or H7 [H7N1(A/turkey/Italy/3889/1999)] virus infection of MDCK cells at different serum dilutions. (**b**) Gating strategy for live NK cells (top panel) and representatives plots identifying CD107a$^+$ (upper left), IFN-γ$^+$ (lower right) or CD107a$^+$ and IFN-γ$^+$ (upper right) populations from enriched CD3$^-$NKp46$^+$ mouse NK cells stimulated with H1^{PR8}-reactive serum antibodies from mice immunized with the indicated vaccines. (**c**) Quantification of activated NK cells after stimulation with H1^{PR8}-reactive serum antibodies from vaccinated mice. Activation was quantified by the percentage of NK cells expressing IFN-γ (left) or CD107a (right). The % CD107a$^+$IFN-γ$^+$ NK cells are shown in Fig. 4f. Mean titers ± SEM (**a**) or individual percentages plus mean ± SEM (**c**) are shown. Significance is calculated using Kruskal-Wallis with Dunn's multiple comparisons test. Each panel presents data derived from n=1 independent experiment. Source data are provided as a Source Data file.

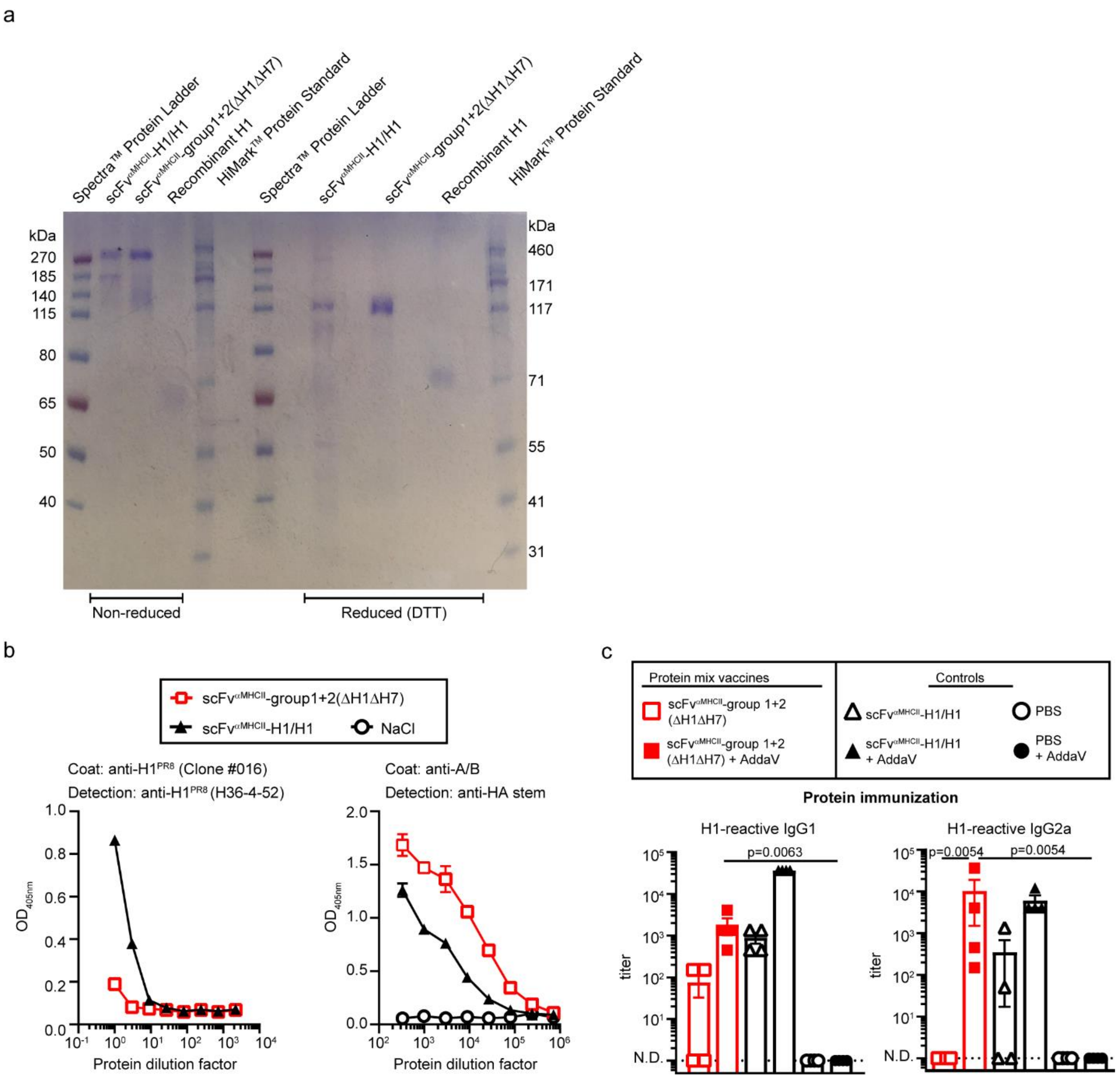

Supplementary Figure 10. Characterization of purified protein vaccines and induction of IgG subclasses in mice vaccinated with protein mix vaccines. a-b HEK293E cells were transfected with a mixture of eight MHCII-targeted HA A (H8, H9, H10, H11, H12, H13, H15 and H16) and eight MHCII-targeted HA B (H2, H3, H4, H5, H6, H14, H17 and H18) plasmids (15.5 µg/plasmid) or with MHCII-targeted A/B bivalent PR8 H1/H1. Vaccine proteins were purified from supernatant using an affinity column with mAb specific for assembled A/B heterodimers. Purified proteins were analyzed (**a**) by SDS page and (**b**) in ELISA with the

indicated antibody combinations (n=3 [H1/H1 protein, NaCl in left panel and mix protein in right panel] or n=2 [mix protein in left panel, NaCl in right panel] technical replicates). **c** Female BALB/cAnNRj mice were immunized twice (week 0 and 5) with the purified MHCII-targeted ΔH1ΔH7 mix protein vaccine or bivalent H1/H1 protein vaccine as control (2.5 µg of purified protein/mouse with or without AddaVax adjuvant, n=4/group). Week 13 sera from vaccinated mice were analyzed for H1-reactive IgG1 (left) and IgG2a (right). Shown are titers for individual mice and mean ± SEM. Significance is calculated using Kruskal-Wallis with Dunn's multiple comparisons test. Each panel presents data derived from n=1 independent experiment. The uncropped gel in **b** and source data for **b** and **c** are provided as a Source Data file.

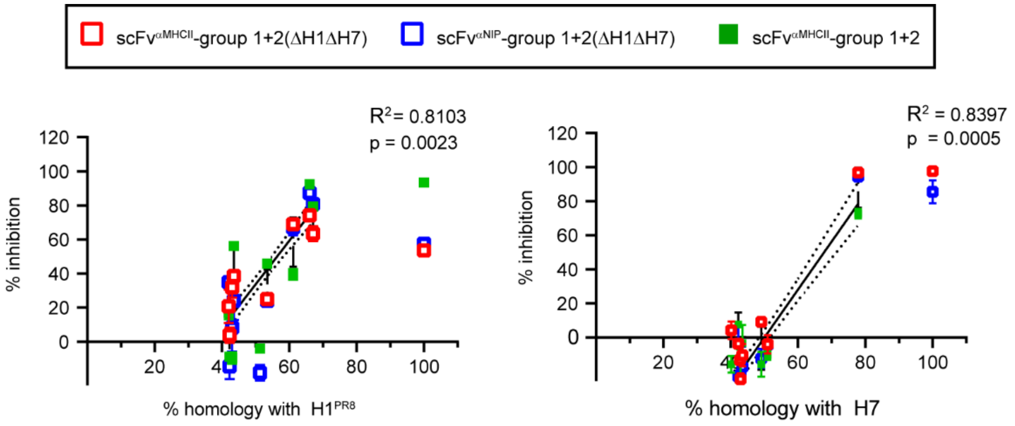

Supplementary Figure 11. Correlation of % inhibition of serum IgG from binding to HA coat (y-axis) to % homology between coat HA and competing HA (x-axis). Female BALB/cAnNRj mice were vaccinated with mix vaccine i.m./EP three times (week 0, 5 and 9, n=8/group) with the indicated mix vaccines. Pooled serum from two weeks after the final vaccination was pre-incubated with soluble recombinant HA proteins (5 µg/ml) prior to analyzing binding of serum IgG to the coat HA in ELISA. Competing HA for the H1 coat: H2 (67% homology), H5 (66%), H6 (61%), H9 (53%), H16 (51%), H3 (44%), H4 (43%), H15 (42%) and H7 (42%). Competing HA for the H7 coat: H15 (77% homology), H14 (51%), H4 (50%), H3 (49%), H1 (42%), H2 (43%), H5 (44%), H16 (40%) and H9 (43%). Analysis was done in technical triplicates. Shown are % homology of the competing HA versus the % inhibition for each triplicate of serum from each of the mix-vaccinated groups (Fig. 5a-b). Correlations were calculated using Pearson correlation coefficients. Shown are R^2 for the Pearson correlation and p values. Source data are provided as a Source Data file.

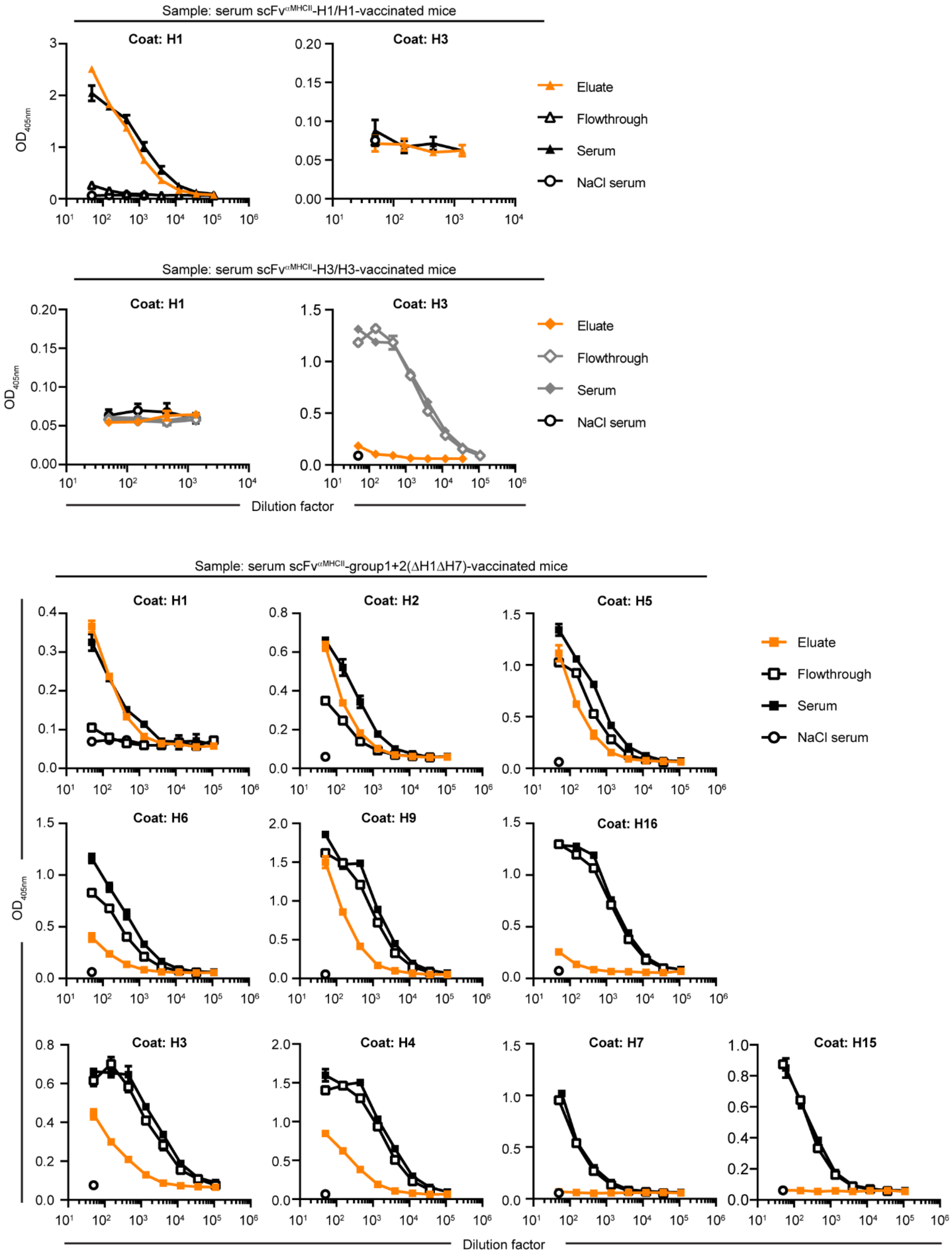
Sample: serum scFvαMHCII-H1/H1-vaccinated mice
Coat: H1
Coat: H3
OD405nm
Eluate
Flowthrough
Serum
NaCl serum
Sample: serum scFvαMHCII-H3/H3-vaccinated mice
Coat: H1
Coat: H3
OD405nm
Eluate
Flowthrough
Serum
NaCl serum
Dilution factor
Sample: serum scFvαMHCII-group1+2(ΔH1ΔH7)-vaccinated mice
Coat: H1
Coat: H2
Coat: H5
Eluate
Flowthrough
Serum
NaCl serum
Coat: H6
Coat: H9
Coat: H16
OD405nm
Coat: H3
Coat: H4
Coat: H7
Coat: H15
Dilution factor

Supplementary Figure 12. H1-reactive antibodies from mix-vaccinated mice crossreact with other HA subtypes. Female BALB/cAnNRj mice were vaccinated thrice (week 0, 5 and 9) with the MHCII-targeted ΔH1ΔH7 mix vaccine i.m./EP (1 µg DNA/plasmid, n=6), or with bivalent MHCII-targeted H1/H1 or H3/H3 (5 µg DNA/plasmid, n=4) as controls. Sera taken two weeks after the third vaccination were pooled. H1-reactive antibodies were affinity purified on $H1^{PR8}$ Sepharose. Total IgG in pooled serum, eluate and flowthrough were analyzed on ELISA coated with the indicated recombinant HA proteins in technical triplicates. Serum from NaCl-vaccinated mice was added on each plate as negative control. Shown are mean ± SD OD_{405nm} of technical triplicates. Each panel presents data derived from n=1 independent experiment. Source data are provided as a Source Data file.

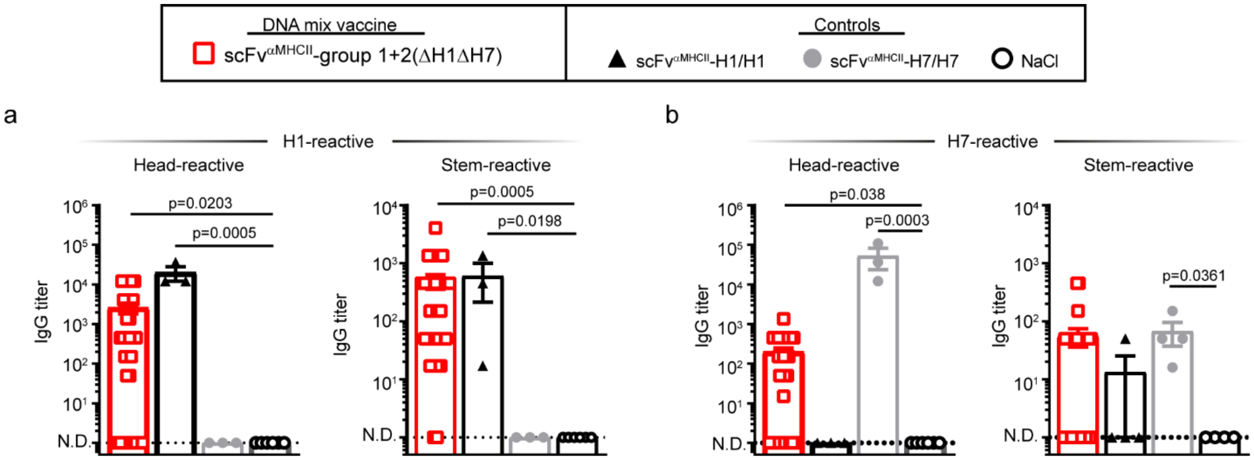

Supplementary Figure 13. Antibody responses against HA head and HA stem domains of H1 and H7. a Female BALB/cAnNRj mice were immunized twice (week 0 and 5) i.m./EP with a scFv$^{\alpha MHCII}$-targeted mix vaccine of 16 HAs (excluding H1 and H7, 1µg DNA/plasmid, n=32). Control immunizations were done with scFv$^{\alpha MHCII}$-targeted vaccines bivalent for H1/H1 or H7/H7 (5 µg DNA/plasmid, n=3) or NaCl (n=6). IgG titers against H1 HA head (left graphs) and stem (right graphs) domains were analyzed in sera four weeks after the second immunization. **b** BALB/c mice were immunized with scFv$^{\alpha MHCII}$-targeted mix vaccine (n=32), bivalent H1/H1 or H7/H7 (n=3/group [left] or n=4/group [right]), or NaCl (n=6 [left] or n=4 [right]) as controls Shown are titers for individual mice and mean ± SEM. Significance is calculated using Kruskal-Wallis with Dunn's multiple comparisons test. Each panel presents data derived from n=1 independent experiment. Source data are provided as a Source Data file.

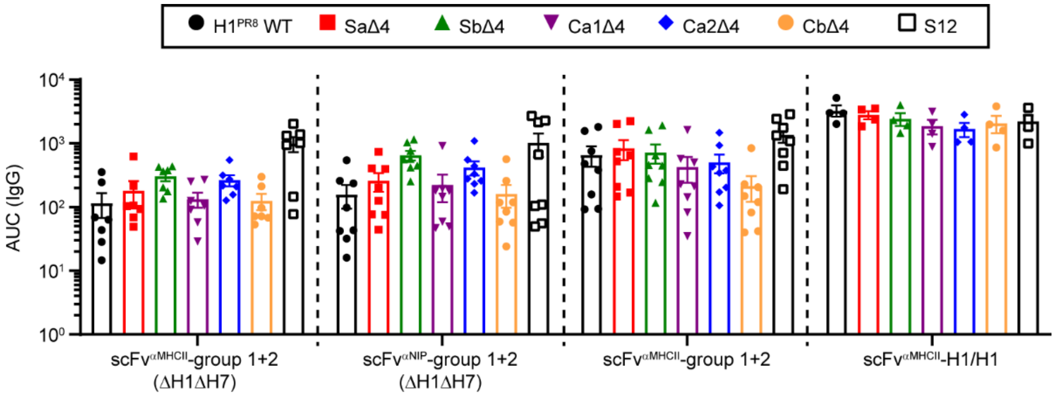

Supplementary Figure 14. Antibody specificity for H1^{PR8} and mutants derived therefrom. Female BALB/cAnNRj mice were immunized thrice (week 0, 5 and 9) i.m./EP with the indicated mix vaccines (1 µg DNA/plasmid, n=7 [scFv$^{\alpha MHCII}$-group1+2(ΔH1ΔH7)] or n=8/group [scFv$^{\alpha NIP}$-group1+2(ΔH1ΔH7) and scFv$^{\alpha MHCII}$-group1+2]), or with scFv$^{\alpha MHCII}$-targeted vaccines bivalent for H1/H1 or H7/H7(n=4) as controls. Individual sera taken two weeks after the third vaccination were analyzed by ELISA for binding of total IgG to recombinant H1^{PR8} as well as various mutants of H1^{PR8} lacking dominant antigenic determinants (mutants are specified in the box). Reactivity of sera to the different H1^{PR8} WT and mutant is shown as AUC for individual mice plus mean + SEM. AUC values obtained with serum from scFv$^{\alpha MHCII}$-targeted H7/H7-vaccinated mice was considered as baseline and was subtracted from AUC values obtained with serum from scFv$^{\alpha MHCII}$-targeted mix- or H1/H1-vaccinated mice. n=1 independent experiment. Source data are provided as a Source Data file.

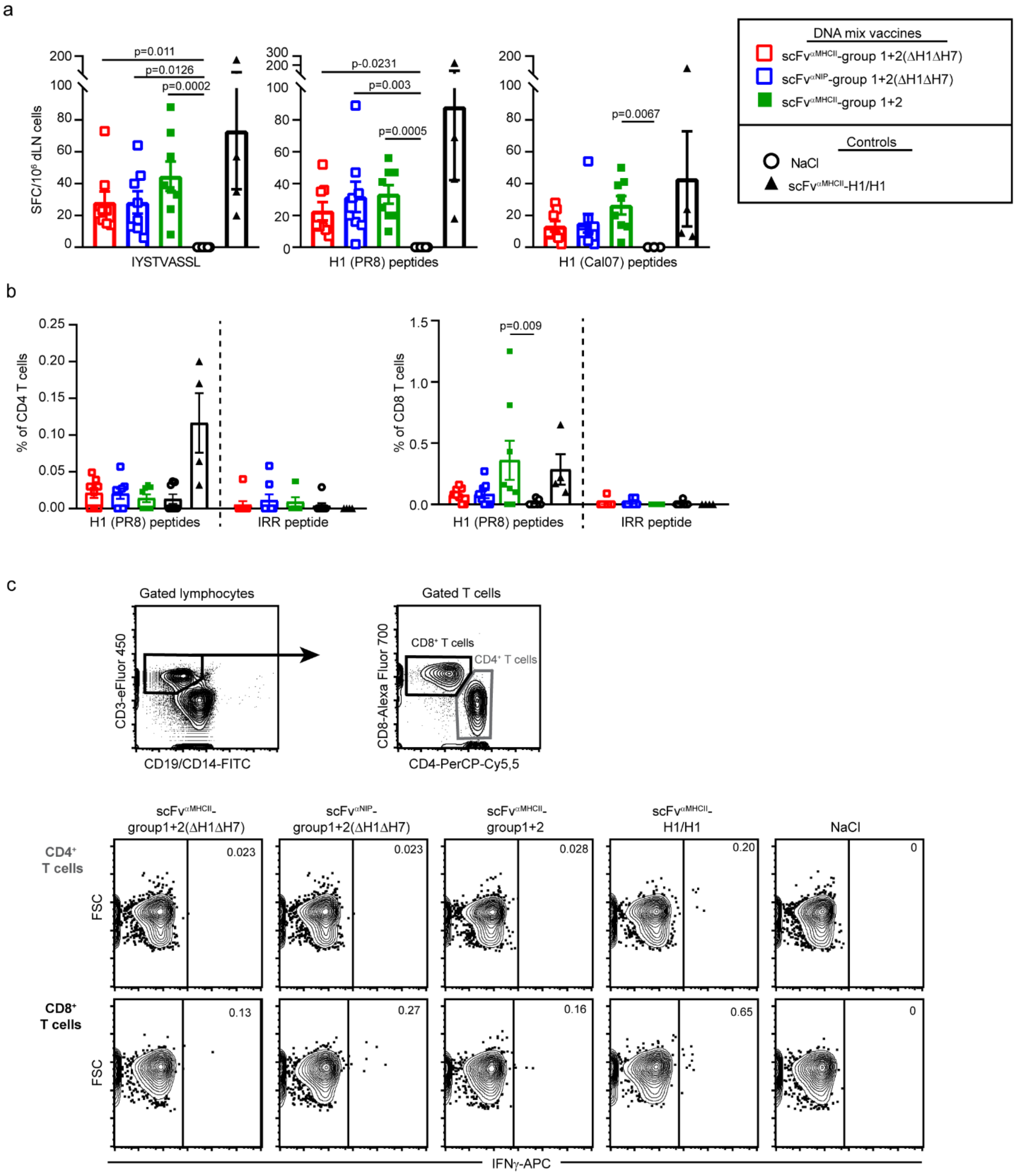

Supplementary Figure 15. Analysis of T cells after vaccination of mice with scFv$^{\alpha MHCII}$-targeted DNA mix vaccine. a Female BALB/cAnNRj mice were vaccinated i.m./EP once with the indicated HA plasmid mix vaccines (1 µg/plasmid, n=8/group), or NaCl (n=8 [for

IYSTVASSL and H1 (PR8) peptide reactivity] or n=3 [for H1 (Cal07) peptide reactivity]). Draining lymph nodes (dLN) were collected 11 days after vaccination, stimulated with H1 peptide IYSTVASSL or overlapping peptides from H1 of PR8 or Cal07, and analyzed for IFN-γ producing cells by ELISPOT. Bivalent scFv$^{\alpha MHCII}$-targeted H1/H1 controls (H1 of PR8, 5µg/plasmid, n=4) are shown for comparison. Shown are spot-forming cells (SFC) per 10^6 cells and mean ± SEM. **b-c** Female BALB/cAnNRj mice were vaccinated i.m./EP three times (week 0, 5 and 9) with the indicated HA plasmid mixes (1µg/plasmid, n=8/group), scFv$^{\alpha MHCII}$-H1/H1 (PR8, 5µg/plasmid, n=4) or NaCl (n=8). Spleens were harvested two weeks after the third immunization and stimulated with overlapping PR8 HA peptides or with an irrelevant peptide (ALWFRNHFVFGGGTKVT from M315 mouse myeloma protein) for twenty hours. (**b**) The frequency of $CD4^+$ and $CD8^+$ IFN-γ producing T cells ($CD14^-CD19^-CD3^+$) was analyzed by flow cytometry. Shown are the % IFN-γ^+ $CD4^+$ and $CD8^+$ T cells. (**c**) Gating strategy for B, and representative examples for analysis of IFN-γ^+ T cell subsets after stimulation with overlapping peptides of PR8 HA. Significance in **a** and **b** is calculated using Kruskal Wallis with Dunn's multiple comparisons test. Each panel presents data derived from n=1 independent experiment. Source data are provided as a Source Data file.

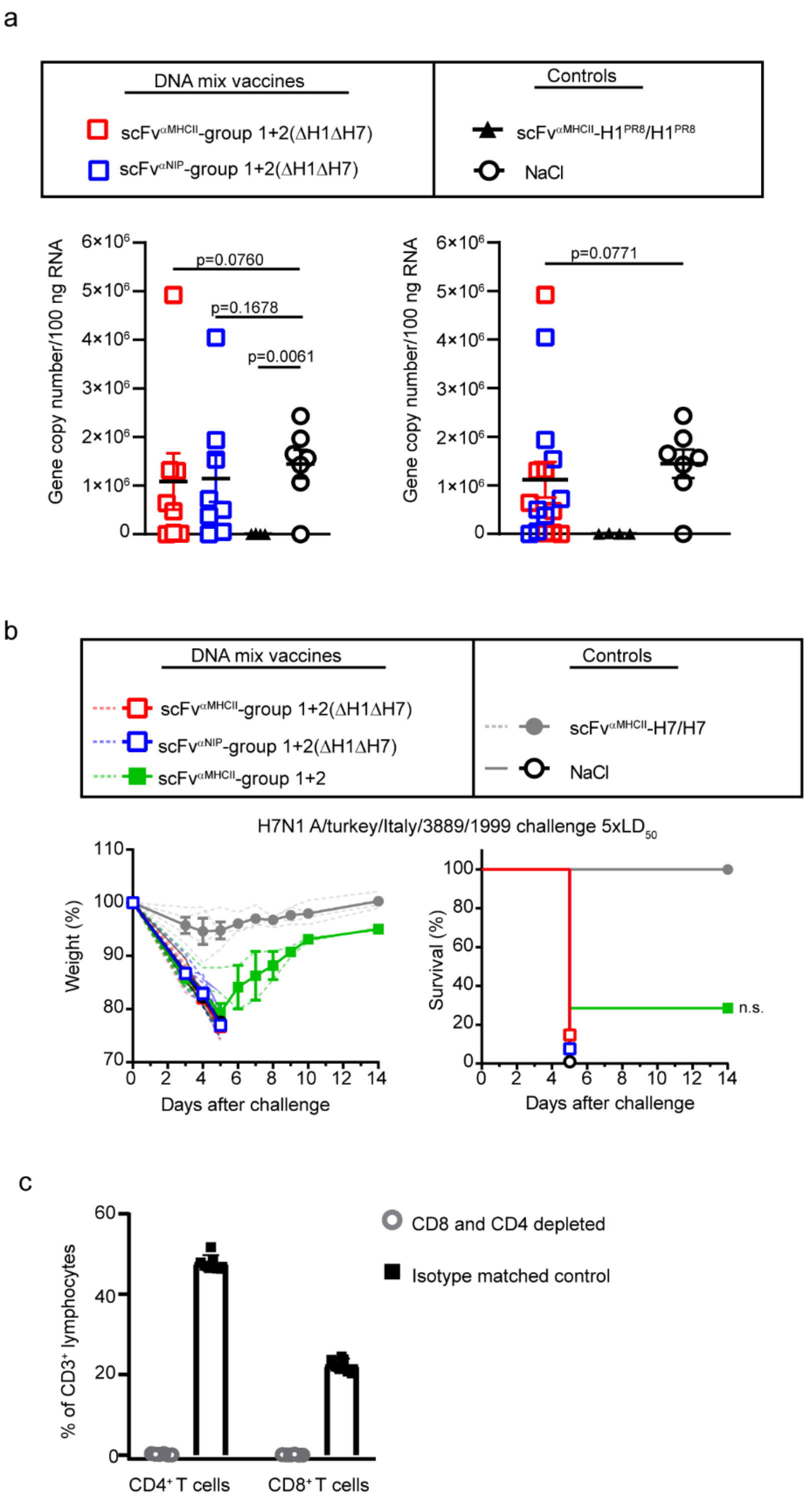

Supplementary Figure 16. Protection against heterologous influenza viruses by the HA mix vaccine. a Female BALB/cAnNRj mice were immunized three times (week 0, 5 and 9) i.m./EP with indicated DNA plasmid mix vaccines (1 µg/DNA plasmid, n=8/group), or with scFV$^{\alpha MHCII}$-

H1/H1 controls (5 µg DNA/plasmid, n=4/group) or NaCl (n=7). Two weeks after final vaccination, mice were infected with 2.5xLD_{50} of H1N1 PR8 virus. At day 5 post challenge, viral loads were determined by qRT-PCR of harvested lung tissue. Gene copy number/100 ng total tissue RNA of individual mice and mean ± SEM are shown. $scFv^{\alpha MHCII}$- and $scFv^{\alpha NIP}$-targeted groups are either shown separately (left) or pooled in one group (right), one-tailed Mann Whitney. **b** Mice were vaccinated as in **a** with indicated DNA plasmid mixes (n=7/group [$scFv^{\alpha MHCII}$-group1+2 and $scFv^{\alpha NIP}$-group1+2(ΔH1ΔH7) or n=8 [$scFv^{\alpha MHCII}$-group1+2(ΔH1ΔH7)], bivalent H7/H7 control (n=4) and or NaCl (N=4). Mice were infected with 5xLD_{50} of H7N1 A/turkey/Italy/3889/1999 virus two weeks after the last vaccination. Weight loss (mean ± SEM, left) and survival (right) are indicated. Mice were euthanized when they reached 80% of their initial weight and removed from the weight curves. At 5xLD_{50}, NaCl-vaccinated mice reach 80% initial weight at day 5-6 when challenged with H7N1 and at day 6-8 when challenges with PR8 virus. Weights of individual mice are indicated in stippled and faded lines. n.s. = not significant, two-tailed Mantel Cox (survival comparing mix vaccines to NaCl) or two-way ANOVA (weight curves). **c** T cell depletion. Mice were immunized twice i.m./EP (week 0 and 5) with the $scFv^{\alpha MHII}$-group1+2(ΔH1ΔH7) mix vaccine (1 µg/plasmid) and treated with anti-CD4 and anti-CD8 (n=16), or isotype matched control (n=13) mAbs every other day from day 26 after the second immunization. Two days later, mice were challenged with 5xLD_{50} of H1N1 virus PR8. Splenocytes were taken from mice euthanized on day 6 and 14 after infection, (n=6 for depleted mice, n=7 for isotype control mice) and cells were stained. Shown are mean % ± SEM of $CD4^+$ and $CD8^+$ T cells of $CD14^-CD19^-CD3^+$ lymphocytes in T cell-depleted and isotype-matched control groups. n=1 independent experiment. Source data are provided as a Source Data file.
