# Supplementary material for: Applying valency-based immuno-selection to generate broadly cross-reactive antibodies against influenza hemagglutinins
Source: Nat Commun. 2024 Feb 12;15:850. doi: 10.1038/s41467-024-44889-w (PMC10861589; doi:10.1038/s41467-024-44889-w)
Supplement: Supplementary file 2 — Reporting Summary [file 41467_2024_44889_MOESM2_ESM.pdf]

nature portfolio

Corresponding author(s): Ranveig Braathen

Last updated by author(s): Dec 21, 2023

Reporting Summary

Nature Portfolio wishes to improve the reproducibility of the work that we publish. This form provides structure for consistency and transparency in reporting. For further information on Nature Portfolio policies, see our Editorial Policies and the Editorial Policy Checklist.

Statistics

For all statistical analyses, confirm that the following items are present in the figure legend, table legend, main text, or Methods section.

n/a	Confirmed	
☐	☒	The exact sample size (*n*) for each experimental group/condition, given as a discrete number and unit of measurement
☐	☒	A statement on whether measurements were taken from distinct samples or whether the same sample was measured repeatedly
☐	☒	The statistical test(s) used AND whether they are one- or two-sided<br>*Only common tests should be described solely by name; describe more complex techniques in the Methods section.*
☒	☐	A description of all covariates tested
☒	☐	A description of any assumptions or corrections, such as tests of normality and adjustment for multiple comparisons
☐	☒	A full description of the statistical parameters including central tendency (e.g. means) or other basic estimates (e.g. regression coefficient) AND variation (e.g. standard deviation) or associated estimates of uncertainty (e.g. confidence intervals)
☐	☒	For null hypothesis testing, the test statistic (e.g. *F*, *t*, *r*) with confidence intervals, effect sizes, degrees of freedom and *P* value noted<br>*Give P values as exact values whenever suitable.*
☒	☐	For Bayesian analysis, information on the choice of priors and Markov chain Monte Carlo settings
☒	☐	For hierarchical and complex designs, identification of the appropriate level for tests and full reporting of outcomes
☐	☒	Estimates of effect sizes (e.g. Cohen's *d*, Pearson's *r*), indicating how they were calculated

Our web collection on statistics for biologists contains articles on many of the points above.

Software and code

Policy information about availability of computer code

Data collection	As described in Methods: The phylogenetic tree was generated using a Clustal Omega multiple alignment of the amino acid sequences of the HA proteins used in this paper. The tree was made with the neighbor-joining method without distance correction (URL: https://www.ebi.ac.uk/Tools/msa/clustalo/ 56). The radial tree was visualized using an online version of TreeDyn 198.3 (URL: http://www.phylogeny.fr/). The graphic amino acid alignment of the HA proteins used in this paper was made using blastp suite (URL: https://blast.ncbi.nlm.nih.gov/Blast.cgi?PROGRAM=blastp&PAGE_TYPE=BlastSearch&LINK_LOC=blasthome).<br>FACS data was collected by Attune NxT flow cytometer (Termo Fischer Scientific and analysed with FlowJo, version 10.8.1 BD Biosciences.<br>ELISpot were councted automatically using an ImmunoSpot® CORE cell counter (Cellular Technology Limited).
Data analysis	ELISA, microneutralization, ADCC, ELISPOT, influenza challenge data resuls were analyzed with GraphPad Prism & Software version (as described in Methods.<br>FACS data were analysed with FlowJo Software, version 10.8.1 BD Biosciences.

For manuscripts utilizing custom algorithms or software that are central to the research but not yet described in published literature, software must be made available to editors and reviewers. We strongly encourage code deposition in a community repository (e.g. GitHub). See the Nature Portfolio guidelines for submitting code & software for further information.

Data

Policy information about availability of data

All manuscripts must include a data availability statement. This statement should provide the following information, where applicable:

- Accession codes, unique identifiers, or web links for publicly available datasets
- A description of any restrictions on data availability
- For clinical datasets or third party data, please ensure that the statement adheres to our policy

Data and material not included in the manuscript of supplementary material will be made available to qualified academic researchers upon request to the corresponding author. The data used to generate the main results shown in the main figures and Supplementary Information are available as Source data. Source data are provided with this paper.

Research involving human participants, their data, or biological material

Policy information about studies with human participants or human data. See also policy information about sex, gender (identity/presentation), and sexual orientation and race, ethnicity and racism.

Reporting on sex and gender	N/A
Reporting on race, ethnicity, or other socially relevant groupings	N/A
Population characteristics	N/A
Recruitment	N/A
Ethics oversight	N/A

Note that full information on the approval of the study protocol must also be provided in the manuscript.

Field-specific reporting

Please select the one below that is the best fit for your research. If you are not sure, read the appropriate sections before making your selection.

- [x] Life sciences
- [ ] Behavioural & social sciences
- [ ] Ecological, evolutionary & environmental sciences

For a reference copy of the document with all sections, see nature.com/documents/nr-reporting-summary-flat.pdf

Life sciences study design

All studies must disclose on these points even when the disclosure is negative.

Sample size	The sample size was decided upon previous studies (Werninghaus et al. Mol Ther. 2023 Jul 5;31(7):2188-2205. doi: 10.1016/j.ymthe.2023.03.012. and Hinke et al. Cell Rep. 2022;39(9):110901. DOI: 10.1016/j.celrep.2022.110901) on similar experiments using 6-8 mice per group to gain statistically significants in various experiments.
Data exclusions	No data was excluded from the study.
Replication	In most experiments 6-8 mice per group were included and the individual mice and mean with SEM is shown. This is clearly described in the figure legend for the respective experiments.
Randomization	We used female BALB/cAnNRj/c mice (Janvier Labs or Taconic Biosciences inc) of the age 6-8 weeks that was randomly put into the various group.
Blinding	The experiments were not performed blinded, but special care was taken to include negative and positive controls in the same experiment.

Reporting for specific materials, systems and methods

We require information from authors about some types of materials, experimental systems and methods used in many studies. Here, indicate whether each material, system or method listed is relevant to your study. If you are not sure if a list item applies to your research, read the appropriate section before selecting a response.

Materials & experimental systems

n/a	Involved in the study
☐	☒ Antibodies
☐	☒ Eukaryotic cell lines
☒	☐ Palaeontology and archaeology
☐	☒ Animals and other organisms
☒	☐ Clinical data
☒	☐ Dual use research of concern
☒	☐ Plants

Methods

n/a	Involved in the study
☒	☐ ChIP-seq
☐	☒ Flow cytometry
☒	☐ MRI-based neuroimaging

Antibodies

Antibodies used

Antibodies for ELISA
Anti-A/B (1 µg/ml, clone 2H11, produced from hybridoma provided by E.L. Reinherz)
Anti-IAV H1N1 (A/Puerto Rico/8/1934) HA (1 µg/ml, clone H36-4-52, produced from hybridoma provided by Siegfried Weiss)
Anti-IAV HA stem (1:3000, clone #2, Cat# 86001-RM01, Sino Biological)
Anti-AIV H3N2 (A/Brisbane/10/2007) HA (1 µg/ml, clone #104, Cat# 11056-R104, Sino Biological)
Anti-rabbit IgG pAb-ALP conjugate, (1:3000, Cat# A3687, Sigma Aldrich)
Anti-mouse IgG pAb-ALP conjugate (1:5000, A2429, Sigma Aldrich)
anti-mouse IgG1[a]-Biotin (1:500, clone10.9, 553500, BD Biosciences)
anti-mouse IgG2a[a]-Biotin (1:500, clone 8.3, 553502, Biosciences)
anti-human IgG-Biotin, (1 µg/ml, clone HP-6017, B3773, Sigma)
anti-IAV nucleoprotein (1 µg/ml, clone HB65, produced from hybridoma H16-L10-4R5, ATCC)

Antibodies for Western blot
Anti-A/B, clone 2H11 (0.33 µg/ml, produced from hybridoma provided by E.L. Reinherz)

Antibodies for Flow cytometry
anti-mouse CD107a (LAMP-1)-PE (2 µg/ml, clone 1D4B, 121612, Biolegend)
anti-mouse CD3e-FITC (5 µg/ml, clone 145-2C11, 35-0031-U500, TONBO reagents)
and NKp46-eFluor 450 (2 µg/ml, clone 29A1.4, 48-3351-82, ThermoFisher Scientific)
anti-mouse IFN-gamma-APC (5 µg/ml, clone XMG1.2, 20-7311-U100, TONBO reagents)

Antibodies for T cell depletion in vivo
Anti-mouse CD4 (100 µg/100 µl, clone GK1.5, produced from hybridoma TIB207, ATCC)
Anti-mouse CD8 (100 µg/100 µl, clone 53-6.72, produced from hybridoma TIB105, ATCC)
Rat IgG2a isotype control (100 µg/100 µl, clone Y13-238, produced from hybridoma CRL-1741, ATCC)
Rat IgG2b isotype control (100 µg/100 µl, clone SFR8-B6, produced from hybridoma HB-152, ATCC)

Validation

Commercially available antibodies have been validated for our applications by their manufacturers. Please see the webpages of the manufacturers for more information.
Anti-A/B, clone 2H11, was first described in Chang et al. (Proc Natl Acad Sci U S A. 1994;91(24):11408-11412 / DOI: 10.1073/pnas.91.24.11408). Binding of anti-A/B to our A/B constructs has been shown in previous publications (Braathen et al. Mol Ther Methods Clin Dev. 2020:17:378-392. DOI: 10.1016 /j.omtm.2020.01.007 and Hinke et al. Cell Rep. 2022;39(9):110901. DOI: 10.1016/j.celrep.2022.110901)
Anti-IAV H1N1 Anti-IAV H1N1 (A/Puerto Rico/8/1934) HA, clone H36-4-52, was first described by Staudt and Gerhard (J Exp Med. 1983;157(2):687-704. DOI: 10.1084/jem.157.2.687. references). Specific binding to H1(PR8) in a DNA plasmid vaccine expressing dimeric proteins has been verified in previous publications (Grødeland et al. J Immunol. 2013;191(6):3221-31. DOI: 10.4049/jimmunol.1300504 and Braathen et al. Mol Ther Methods Clin Dev. 2020:17:378-392. DOI: 10.1016 /j.omtm.2020.01.007)
Efficiency of Anti-mouse CD4, clone GK1.5, and anti-mouse CD8, clone 53-6.72 for T cell depletion in BALB/c mice was verified in a previous publication (Grødeland et al. J Immunol. 2013;191(6):3221-31. DOI: 10.4049/jimmunol.1300504), as well as in this manuscript.

Eukaryotic cell lines

Policy information about cell lines and Sex and Gender in Research

Cell line source(s)

Cell lines used are MDCK (RRID:CVCL0422, cat# CCL-34) and HEK293E (RRID:CVCL0045, cat# CRL-1573) both from ATCC,Manassas, USA.

Authentication

None of the cell lines used were authenticated.

Mycoplasma contamination	Cell lines were tested for mycoplasma contamination.
Commonly misidentified lines (See ICLAC register)	No commonly misidentified cell lines were used in the study.

Animals and other research organisms

Policy information about studies involving animals; ARRIVE guidelines recommended for reporting animal research, and Sex and Gender in Research

Laboratory animals	We used female BALB/cAnNRj/c, 6-8 week age at starting point for the experiments. Mice were housed in standard cages with an MDU environment with a 12-hour light/dark cycle at a room temperature of 22°C ±2°C, humidity of 50%±5%, with free access to food and water.
Wild animals	The study did not involve wild animals.
Reporting on sex	Used only female mice
Field-collected samples	The study did not include samples collected from the field.
Ethics oversight	All experiments were reviewed and approved by the Norwegian Animal Research Authority and were carried out in accordance with the recommendations from the Guide for the Care and Use of Laboratory Animals of the Norwegian National Institute of Health.

Note that full information on the approval of the study protocol must also be provided in the manuscript.

Plants

Seed stocks	N/A
Novel plant genotypes	N/A
Authentication	N/A

Flow Cytometry

Plots

Confirm that:

- [x] The axis labels state the marker and fluorochrome used (e.g. CD4-FITC).
- [x] The axis scales are clearly visible. Include numbers along axes only for bottom left plot of group (a 'group' is an analysis of identical markers).
- [x] All plots are contour plots with outliers or pseudocolor plots.
- [x] A numerical value for number of cells or percentage (with statistics) is provided.

Methodology

Sample preparation	Spleens were harvested from naive BLAB/c mice and dissociated in gentleMACS™ C Tubes . Splenocytes were treated with Tris-buffered ammonium chloride for 5 min on ice. Cells were filtered through a 70 nm Nylon strainer before counting. For analysis of activated NK cells, NK cell were isolated using an NK cell negative selection kit (130-115-818, Milteny Biotech) according to the manufacturers protocol.
Instrument	Attune NxT flow cytometer (Termo Fischer Scientific)
Software	FlowJo, version 10.8.1 BD Biosciences.
Cell population abundance	NK cell populations in fresh mouse spleens were determined before and after NK negative selection by staining samples with anti-mouse Nkp46 and anti-mouse CD3e and analysis in flow cytometry. NK population in fresh mouse spleen was around 6% before enrichment. After enrichment, the abundance of NK cells was around 80%.
Gating strategy	Gating of activated NK cells: Mouse splenocytes were gated for lymphocytes by size and granularity on SSC-A vs FSC-A. Single lymphocytes cells were gated on FSC-H vs FSC-A, NK cells gated on CD3e vs NKp46 (NK cells are CD3-NKp46+), and live NK cells gated on SSC-A vs Live/Dead (Ghost dye, Tonbo Reagents), and the live NK cells were plotted on CD107a vs IFN-gamma.

Positive NK cells were identified as CD107+ or IFN-gamma+ single postive, or CD107+IFN-gamma+ double positive live NK cells. The positive and negative gating was determined by OneCome eBeads™ stained with the individual flourochrom antibodies used in each experiment.

☒ Tick this box to confirm that a figure exemplifying the gating strategy is provided in the Supplementary Information.
